# Comparison of co-expression measures: mutual information, correlation, and model based indices

**DOI:** 10.1186/1471-2105-13-328

**Published:** 2012-12-09

**Authors:** Lin Song, Peter Langfelder, Steve Horvath

**Affiliations:** 1 Human Genetics, David Geffen School of Medicine, University of California, California, Los Angeles, USA; 2 Biostatistics, School of Public Health, University of California, California, Los Angeles, USA

## Abstract

**Background:**

Co-expression measures are often used to define networks among genes. Mutual information (MI) is often used as a generalized correlation measure. It is not clear how much MI adds beyond standard (robust) correlation measures or regression model based association measures. Further, it is important to assess what transformations of these and other co-expression measures lead to biologically meaningful modules (clusters of genes).

**Results:**

We provide a comprehensive comparison between mutual information and several correlation measures in 8 empirical data sets and in simulations. We also study different approaches for transforming an adjacency matrix, e.g. using the topological overlap measure. Overall, we confirm close relationships between MI and correlation in all data sets which reflects the fact that most gene pairs satisfy linear or monotonic relationships. We discuss rare situations when the two measures disagree. We also compare correlation and MI based approaches when it comes to defining co-expression network modules. We show that a robust measure of correlation (the biweight midcorrelation transformed via the topological overlap transformation) leads to modules that are superior to MI based modules and maximal information coefficient (MIC) based modules in terms of gene ontology enrichment. We present a function that relates correlation to mutual information which can be used to approximate the mutual information from the corresponding correlation coefficient. We propose the use of polynomial or spline regression models as an alternative to MI for capturing non-linear relationships between quantitative variables.

**Conclusion:**

The biweight midcorrelation outperforms MI in terms of elucidating gene pairwise relationships. Coupled with the topological overlap matrix transformation, it often leads to more significantly enriched co-expression modules. Spline and polynomial networks form attractive alternatives to MI in case of non-linear relationships. Our results indicate that MI networks can safely be replaced by correlation networks when it comes to measuring co-expression relationships in stationary data.

## Background

Co-expression methods are widely used for analyzing gene expression data and other high dimensional “omics” data. Most co-expression measures fall into one of two categories: correlation coefficients or mutual information measures. MI measures have attractive information-theoretic interpretations and can be used to measure non-linear associations. Although MI is well defined for discrete or categorical variables, it is non-trivial to estimate the mutual information between quantitative variables, and corresponding permutation tests can be computationally intensive. In contrast, the correlation coefficient and other model based association measures are ideally suited for relating quantitative variables. Model based association measures have obvious statistical advantages including ease of calculation, straightforward statistical testing procedures, and the ability to include additional covariates into the analysis. Researchers trained in statistics often measure gene co-expression by the correlation coefficient. Computer scientists, trained in information theory, tend to use a mutual information (MI) based measure. Thus far, the majority of published articles use the correlation coefficient as co-expression measure [1-5] but hundreds of articles have used the mutual information (MI) measure [6-12].

Several articles have used simulations and real data to compare the two co-expression measures when clustering gene expression data. Allen et al. have found that correlation based network inference method WGCNA [5] and mutual information based method ARACNE [9] both perform well in constructing global network structure [13]; Steuer et al. show that mutual information and the Pearson correlation have an almost one-to-one correspondence when measuring gene pairwise relationships within their investigated data set, justifying the application of Pearson correlation as a measure of similarity for gene-expression measurements [14]. In simulations, no evidence could be found that mutual information performs better than correlation for constructing co-expression networks [15]. However, MI continues to be used in recent publications. Some authors have argued that MI is more robust than Pearson correlation in terms of distinguishing various clustering solutions [10]. Given the debates, it remains an open question whether mutual information could be supplanted by standard model based association measures. We affirmatively answer this question by i) reviewing the close relationship between mutual information and likelihood ratio test statistic in the case of categorical variables, ii) finding a close relationship between mutual information and correlation in simulations and empirical studies, and iii) proposing polynomial and spline regression models as alternatives to mutual information for modeling non-linear relationships.

While previous comparisons involved the Pearson correlation, we provide a more comprehensive comparison that considers i) different types of correlation coefficients, e.g. the biweight midcorrelation (bicor), ii) different approaches for constructing MI based and correlation based networks, iii) different ways of transforming a network adjacency matrix (e.g. the topological overlap reviewed below [4,16-18]), and iv) 8 diverse gene expression data from yeast, mouse and humans. Our unbiased comparison evaluates co-expression measures at the level of gene pair relationships and at the level of forming co-expression modules (clusters of genes).

This article presents the following results. First, probably the most comprehensive empirical comparison to date is used to evaluate which pairwise association measure leads to the biologically most meaningful network modules (clusters) when it comes to functional enrichment with GO ontologies. Second, polynomial regression and spline regression methods are evaluated when it comes to defining non-linear association measures between gene pairs. Third, simulation studies are used to validate a functional relationship (cor-MI function) between correlation and mutual information in case that the two variables satisfy a linear relationship. Our comprehensive empirical studies illustrate that the cor-MI function can be used to approximate the relationship between mutual information and correlation in case of real data sets which indicates that in many situations the MI measure is not worth the trouble. Gene pairs where the two association measures disagree are investigated to determine whether technical artifacts lead to the incongruence.

Overall, we find that bicor based co-expression measure is an attractive co-expression measure, particularly when limited sample size does not permit the detection of non-linear relationships. Our theoretical results, simulations, and 8 different gene expression data sets show that MI is often inferior to correlation based approaches in terms of elucidating gene pairwise relationships and identifying co-expression modules. A signed correlation network transformed via the topological overlap matrix transformation often leads to the most significant functional enrichment of modules. Polynomial and spline regression model based statistical approaches are promising alternatives to MI for measuring non-linear relationships.

### Association measure and network adjacency

An association measure is used to estimate the relationships between two random variables. For example, correlation is a commonly used association measure. There are different types of correlations. While the Pearson correlation, which measures the extent of a linear relationship, is the most widely used correlation measure, the following two more robust correlation measures are often used. First, the Spearman correlation is based on ranks, and measures the extent of a monotonic relationship between *x* and *y*. Second, “bicor” (refer to Materials and Methods for definition and details) is a median based correlation measure, and is more robust than the Pearson correlation but often more powerful than the Spearman correlation [19,20]. All correlation coefficients take on values between −1 and 1 where negative values indicate an inverse relationship. A correlation coefficient is an attractive association measure since i) it can be easily calculated, ii) it affords several asymptotic statistical tests (regression models, Fisher transformation) for calculating significance levels (p-values), and iii) the sign of correlation allows one to distinguish between positive and negative relationships. Other association measures, such as mutual information, will be introduced in the next sections.

Association measures can be transformed into network adjacencies. For *n* variables *v*_1_,…,*v*_*n*_, an adjacency matrix *A *= (*A*_*ij*_) is an *n *×* n *matrix quantifying the pairwise connection strength between variables. An (undirected) network adjacency satisfies the following conditions:

(1) $$\begin{array}{rcl} 0\leq A_{\text{ij}} & \leq & 1,\\ A_{\text{ij}} & = & A_{\text{ji}},\\ A_{\text{ii}} & = & 1. \end{array}$$

An association network is defined as a network whose nodes correspond to random variables and whose adjacency matrix is based on the association measure between pairs of variables [21]. Association networks describe the pair wise associations between variables (interpreted as nodes). For a given set of nodes, there is a one-to one relationship between the association network and the adjacency matrix. In order to build an association network for *n* variables *v *= (*v*_1_,…,*v*_*n*_), we start by defining an association measure *AssocMeasure*(*xy*) as a real valued function of two vectors *x*, *y*. We then apply this function on the set of *N *=* n*^2^variable pairs {*Pair*_1 _= (*v*_1_*v*_1_),*Pair*_2 _= (*v*_1_*v*_2_),…,*Pair*_*N *_= (*v*_*N*_*v*_*N*_)}, resulting in an *n *×* n *dimensional matrix

(2) $$S=(\text{AssocMeasure}(v_{i},v_{j})).$$

Then, one needs to specify how the association matrix *S* is transformed into an adjacency matrix. This involves three steps: 1) symmetrize *S* ; 2) transform (and/or threshold) *S* to [0,1] ; 3) set diagonal values to 1. As for step 1, many methods can be used to symmetrize *S* if it is non-symmetric, such as the following three ways:

(3) $$\begin{array}{l} S_{\text{ij}}^{\text{min}}\;=\;\text{min}(S_{\text{ij}},S_{\text{ji}}) \end{array}$$

(4) $$\begin{array}{l} S_{\text{ij}}^{\mathrm{ave}}\;=\;\frac{S_{\text{ij}}+S_{\text{ji}}}{2} \end{array}$$

(5) $$\begin{array}{l} S_{\text{ij}}^{\text{max}}\;=\;\text{max}(S_{\text{ij}},S_{\text{ji}}). \end{array}$$

As for step 2, if *LowerBounds*(*S*) and *UpperBounds*(*S*) denote symmetric matrices of element-wise lower and upper bounds for *S*, then a simple transformation can be defined as:

(6) $$A={\left(\frac{S-\text{LowersBound}(S)}{\text{UpperBounds}(S)-\text{LowerBound}(S)}\right)}^{\beta },$$

where the power *β* is constant and denotes a soft threshold. As an example, assume that the association measure is given by a correlation coefficient, i.e. *S *= (*cor*(***x***_*i*_,***x***_*j*_)). Since each correlation has the lower bound −1 and upper bound + 1 , Eq. 6 reduces to the case of a signed weighted correlation network given by [4,22]:

(7) $$A_{\text{ij}}={\left(\frac{1+\text{cor}(x_{i},x_{j})}{2}\right)}^{\beta }.$$

Additional details of correlation based adjacencies (unweighted or weighted, unsigned or signed) are described in Materials and Methods.

#### Network adjacency based on co-expression measures

When dealing with gene expression data, *x*_*i *_denotes the expression levels of the i-th gene (or probe) across multiple samples. In this article, we assume that the *m* components of *x*_*i *_correspond to random independent samples. Co-expression measures can be used to define co-expression networks in which the nodes correspond to genes. The adjacencies *A*_*ij*_ encode the similarity between the expression profiles of genes *i* and *j*. In practice, transformations such as the topological overlap measure (TOM) [4,16-18] are often used to turn an original network adjacency matrix into a new one. Details of TOM transformation are reviewed in Materials and Methods.

### Mutual information networks based on categorical variables

Assume two random samples *dx* and *dy* of length *m* from corresponding discrete or categorical random variables *DX* and *DY*. Each entry of *dx* equals one of the following *R* levels *ldx*_1_,…,*ldx*_*R*_. The mutual information (MI) is defined as:

(8) $$\text{MI}(\text{dx},\text{dy})=\overset{R_{x}}{\underset{r=1}{\sum }}\overset{R_{y}}{\underset{c=1}{\sum }}p(\text{ld}x_{r},\text{ld}y_{c})\log\left(\frac{p(\text{ld}x_{r},\text{ld}y_{c})}{p(\text{ld}x_{r})p(\text{ld}y_{c})}\right)$$

where *p*(*ldx*_*r*_) is the frequency of level *r* of *dx*, and log is the natural logarithm. Note that the following **simple relationship exists between the mutual information (Eq. 8) and the likelihood ratio test statistic** (described in Additional file 1):

(9) $$\text{MI}(\text{dx},\text{dy})=\frac{\text{LRT.statistic}(\text{dx},\text{dy})}{2m}$$

This relationship has many applications. First, it can be used to prove that the mutual information takes on non-negative values. Second, it can be used to calculate an asymptotic p-value for the mutual information. Third, it points to a way for defining a mutual information measure that adjusts for additional conditioning variables *z*_1_,*z*_2_,… Specifically, one can use a multivariate *multinomial regression model* for regressing *dy* on *dx* and the conditioning variables. Up to a scaling factor of 2*m*, the likelihood ratio test statistic can be interpreted as a (non-symmetric) measure of mutual information between *dx* and *dy* that adjusts for conditioning variables. More detailed discussion of mutual information can be found in [14,23,24]. In Additional file 1, we describe association measures between categorical variables in detail, including LRT statistic and MI.

As discussed below, numerous ways have been suggested for construct an adjacency matrix based on MI. Here we describe an approach that results in a weighted adjacency matrix. Consider *n* categorical variables *dx*_1_,*dx*_2_,…,*dx*_*n*_. Their mutual information matrix *MI*(*dx*_*i*_,*dx*_*j*_) is a similarity matrix *S* whose entries are bounded from below by 0. To arrive at an upper bound, we review the relationship between mutual information and entropy (the following equation is text book knowledge):

(10) MI(dx,dy)=Entropy(dx)+Entropy(dy)−Entropy(dx,dy)

where *Entropy*(*dx*) denotes the entropy of *dx* and *Entropy*(*dx*,*dy*) denotes the joint entropy (refer to Additional file 1). Using Eq. 10, one can prove that the mutual information has the following 3 upper bounds:

(11) $$\begin{array}{ccc} \text{MI}(\text{dx},\text{dy}) & \leq & \text{min}(\text{Entropy}(\text{dx}),\text{Entropy}(\text{dy})), \end{array}$$

(12) $$\begin{array}{ccc} \text{MI}(\text{dx},\text{dy}) & \leq & \frac{\text{Entropy}(\text{dx})+\text{Entropy}(\text{dy})}{2}, \end{array}$$

(13) $$\begin{array}{ccc} \text{MI}(\text{dx},\text{dy}) & \leq & \text{max}(\text{Entropy}(\text{dx}),\text{Entropy}(\text{dy})). \end{array}$$

Using Eq. 6 with *β *= 1, lower bounds of 0 and *UpperBounds*_*ij *_= (*Entropy*(*dx*_*i*_) + *Entropy*(*dx*_*j*_))/2 (Eq. 12) results in the *symmetric uncertainty based mutual information adjacency matrix*:

(14) $$A_{\text{ij}}^{\text{MI},\text{SymmetricUncertainty}}=\frac{2\text{MI}(dx_{i},dx_{j})}{\text{Entropy}(dx_{i})+\text{Entropy}(dx_{j})}.$$

A transformation of *A*^*MI*,*SymmetricUncertainty *^leads to the *universal mutual information based adjacency matrix version 1* (denoted AUV1):

(15) $$A_{\text{ij}}^{\text{MI},\text{UniversalVersion}1}=\frac{A_{\text{ij}}^{\text{MI},\text{SymmetricUncertainty}}}{2-A_{\text{ij}}^{\text{MI},\text{SymmetricUncertainty}}}$$

One can easily prove that $0\leq A_{\text{ij}}^{\text{MI},\text{UniversalVersion}1}\leq 1$. The term “universal” reflects the fact that the adjacency based dissimilarity ${\text{dissMI}}_{\text{ij}}^{\text{UniveralVersion}1}=1-A^{\text{MI},\text{UniversalVersion}1}$ turns out to be a universal distance function [25]. Roughly speaking, the universality of ${\text{dissMI}}_{\text{ij}}^{\text{UniveralVersion}1}$ implies that any other distance measure between *dx*_*i *_and *dx*_*j*_ will be small if $\text{disM}I_{\text{ij}}^{\text{UniveralVersion}1}$ is small. The term “distance” reflects the fact that *dissMI*^*UniveralVersion*1 ^satisfies the properties of a distance including the triangle inequality.

Another adjacency matrix is based on the upper bound implied by inequality 13. We define the *universal mutual information based adjacency matrix version 2*, or AUV2, as follows:

(16) $$A^{\text{MI},\text{UniversalVersion}2}=\frac{\text{MI}(dx_{i},dx_{j})}{\text{max}(\text{Entropy}(dx_{i}),\text{Entropy}(dx_{j}))}.$$

The name reflects the fact that *dissMI*^*UniveralVersion*2 ^= 1−*A*^*MI*,*UniversalVersion*2^ is also a universal distance measure [25]. While *A*^*MI*,*UniversalVersion*1 ^and *A*^*MI*,*UniversalVersion*2^ are in general different, we find very high Spearman correlations (*r *> 0*.*9 ) between their vectorized versions.

Many alternative approaches exist for defining MI based networks, e.g. ARACNE [9], CLR [26], MRNET [27] and RELNET [6,28] are described in Materials and Methods.

### Mutual information networks based on discretized numeric variables

In its original inception, the mutual information measure was only defined for discrete or categorical variables, see e.g. [23]. It is challenging to extend the definition to *quantitative* variables. But, several strategies have been proposed in the literature [7,28,29]. In this article, we will only consider the following approach which is based on discretizing the numeric vector *x* by using the equal width discretization method. This method partitions the interval [*min*(*x*),*max*(*x*)] into equal-width bins (sub-intervals). The vector *discretize*(*x*) has the same length as *x* but its *l*-th component reports the bin number in which *x*_*l*_ falls:

(17) $$dx_{l}=\text{discretize}{\left(x\right)}_{l}=r\;\text{if}\;x_{l}\in \text{bi}n_{r}.$$

The number of bins, *no.bins*, is the only parameter of the equal-width discretization method.

In our subsequent studies, we calculate an MI-based adjacency matrix using the following three steps. First, numeric vectors of gene expression profiles are discretized according to the equal-width discretization method with the default number of bins given by $\text{no.bins}=\sqrt{m}$. Second, the mutual information *MI*_*ij *_=* MI*(*discretize*(*x*_*i*_),*discretize*(*x*_*j*_)) is calculated between the discretized vectors based on Eq. 10 and the Miller Madow entropy estimation method (detailed in Additional file 1). Third, the MI matrix is transformed into one of three possible MI-based adjacency matrices: *A*^*MI*,*SymmetricUncertainty*^ (Eq. 14), *A*^*MI*,*UniversalVersion*1^ (Eq. 15), *A*^*MI*,*UniversalVersion*2 ^(Eq. 16).

## Results

### An equation relating *MI*(*discretize*(*x*),*discretize*(*y*)) to *cor*(*x*,*y*)

As described previously, the mutual information *MI*(*discretize*(*x*),*discretize*(*y*)) between the discretized vectors can be used as an association measure. Note that *MI*(*discretize*(*x*),*discretize*(*y*)) is quite different from *cor*(*xy*) in the following aspects. First, the estimated mutual information depends on parameter choices, e.g. the number of bins used in the equal-width discretization step for defining *dx *=* discretize*(*x*). Second, the mutual information aims to measure general dependence-relationships while the correlation only measures linear or monotonic relationships. Third, the equations for the two measures are very different. Given these differences, it is surprising that a simple approximate relationship holds between the two association measures if *xy* are samples from a bivariate normal distribution and the equal-width discretization method is used with $\text{no.bins}=\sqrt{m}$. Under these assumptions, we will show that *A*^*MI*,*UniversalVersion*2 ^can be accurately approximated as follows:

(18) $$\begin{array}{lll} \;A^{\text{MI},\text{UniversalVersion}2}(\text{dx},\text{dy}) & =\frac{\text{MI}(\text{dx},\text{dy})}{\text{max}(\text{Entropy}(\text{dx}),\text{Entropy}(\text{dy}))}\; & \;\\ & \approx F^{\text{cor}-\text{MI}}(\text{cor}(x,y)),\; \end{array}$$

where the “cor-MI” function [21]

(19) $$F^{\text{cor}-\text{MI}}(s)=\frac{\log(1+\varepsilon -s^{2})}{\log(\varepsilon )}(1-\omega )+\omega$$

depends on the following two parameters

(20) $$\begin{array}{l} \omega =0.43m^{-0.30}\\ \varepsilon =\omega^{2.2}\;. \end{array}$$

In general, one can easily show that *F*^*cor*−*MI*^(*s*) is a monotonically increasing function that maps the unit interval [0,1] to [0,1] if the two parameters *ω* and *ε* satisfy the following relationship

(21) 0<ε≤ω<1.

Eq. 18 was stated in terms of the Pearson correlation, but it also applies for bicor as can be seen from our simulation studies.

### Simulations where *x* and *y* represent samples from a bivariate normal distribution

**Figure 1:**
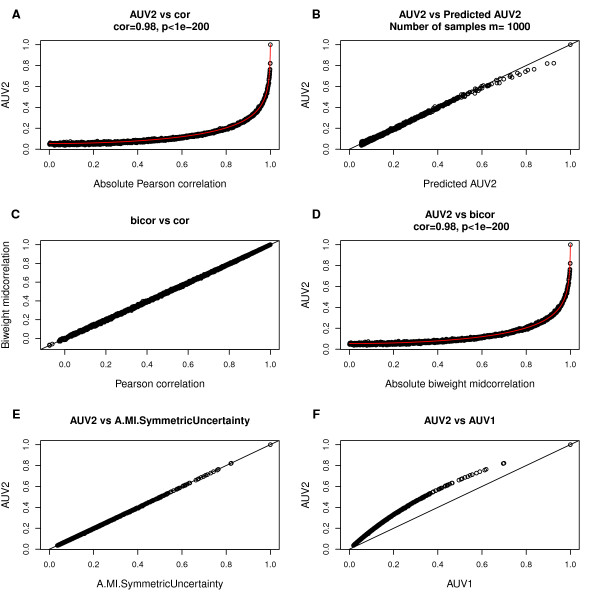
**Relating mutual information based adjacencies to the Pearson correlation and biweight midcorrelation in simulation.** Each point corresponds to a pair of numeric vectors *x* and *y* with length *m *= 1000 . These pairs of vectors were simulated to exhibit different correlations. AUV1, AUV2, cor, bicor are abbreviations for *A*^*MI*,*UniversalVersion*1^, *A*^*MI*,*UniversalVersion*2^, Pearson correlation and biweight midcorrelation, respectively. **(A)** MI-based adjacency *A*^*MI*,*UniversalVersion*2 ^versus absolute Pearson correlation. Spearman correlation of the two measures and the corresponding p-value are shown at the top, implying a strong monotonic relationship. The red line shows the predicted *A*^*MI*,*UniversalVersion*2 ^according to *F*^*cor*−*MI*^(Eq. 18). Note that the prediction function is highly accurate in simulation. **(B)** Observed *A*^*MI*,*UniversalVersion*2 ^versus its predicted value. The straight line has slope 1 and intercept 0. **(C)** Observed Pearson correlation (x-axis) and the corresponding bicor values (y-axis). The straight line has slope 1 and intercept 0. These 2 measurements are practically indistinguishable when x and y are normally distributed. **(D)*** A*^*MI*,*UniversalVersion*2 ^versus bicor. Spearman correlation and p-value of the 2 measurements are presented at the top, and predicted *A*^*MI*,*UniversalVersion*2 ^are shown as the red line. **(E)*** A*^*MI*,*UniversalVersion*2 ^versus *A*^*MI.SymmetricUncertainty*^. **(F)*** A*^*MI*,*UniversalVersion*2 ^versus *A*^*MI*,*UniversalVersion*1^.

Here we use simulation studies to illustrate that *F*^*cor*−*MI *^(Eq. 19) can be used for predicting or approximating *A*^*MI*,*UniversalVersion*2 ^from the corresponding correlation coefficients (Eq. 18). Specifically, we simulate 2000 pairs of sample vectors *x* and *y* from a bivariate normal distribution. Each pair of vectors *x* and *y* is simulated to exhibit different pairwise correlations. Figure 1 shows the relationships of the MI-based adjacency measures with the (observed) Pearson correlation (cor) or biweight midcorrelation (bicor) when each of the vectors contains *m *= 1000 components but the relationship has been confirmed for *m* ranging from 20 to 10000. As can be seen from Figures (1A, B), the cor-MI function (Eq. 18) with parameters specified in Eq. 20 provides a highly accurate prediction of *A*^*MI*,*UniversalVersion*2^ (Eq. 16) on the basis of *cor*(*x*,*y*) and *m*. Since *x* and *y* are normally distributed, the Pearson correlation and bicor are practically indistinguishable (Figure 1C). Thus, replacing cor by bicor leads to equally good predictions of *A*^*MI*,*UniversalVersion*2^ (Figure 1D). Figure (1E) shows that *A*^*MI*,*UniversalVersion*2^ is practically indistinguishable from *A*^*MI*,*SymmetricUncertainty*^. This suggests that cor-MI function can also be used to predict *A*^*MI*,*SymmetricUncertainty*^ on the basis of the correlation measure. Figure (1F) indicates that *A*^*MI*,*UniversalVersion*1^and *A*^*MI*,*UniversalVersion*2^ are different from each other but satisfy a monotonically increasing relationship.

### Empirical studies involving 8 gene expression data sets

Our simulation results show that both the robust biweight midcorrelation and the Pearson correlation can be used as input of *F*^*cor*−*MI*^ for predicting *A*^*MI*,*UniversalVersion*2 ^when the underlying variables satisfy pairwise bivariate normal relationships. However, it is not clear whether *F*^*cor*−*MI*^ can also be used to relate correlation and mutual information in real data applications. In this section, we report 8 empirical studies to study the relationship between MI and the robust correlation measure bicor. To focus the analysis on genes that are likely to reflect biological variation and to reduce computational burden, we selected the 3000 genes with highest variance across the microarray samples for each data set. Description of data sets can be found in Materials and Methods.

**Figure 2:**
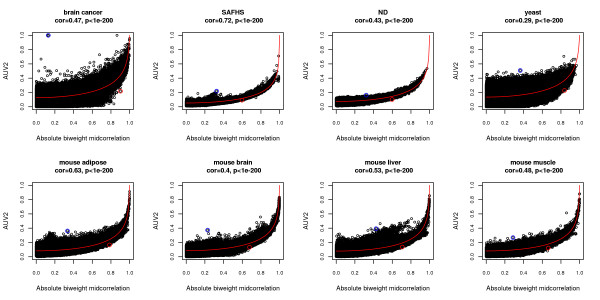
**Comparison of correlation and mutual information based co-expression measures in 8 empirical data sets.** Absolute value of bicor versus *A*^*MI*,*UniversalVersion*2 ^for all probe pairs in each data set. The Spearman correlation and corresponding p-value between the two measures are shown at the top. The two measures show different levels of monotonic relationships in data sets. The red curve predicts *A*^*MI*,*UniversalVersion*2 ^from bicor based on Eq. 18. The blue circle highlights the probe pair with the highest *A*^*MI*,*UniversalVersion*2 ^z-score among those with insignificant bicor z-scores (less than 1*.*9 ); the red circle highlights the probe pair with the highest bicor z-score among those with insignificant *A*^*MI*,*UniversalVersion*2 ^z-scores (less than 1*.*9 ).

**Figure 3:**
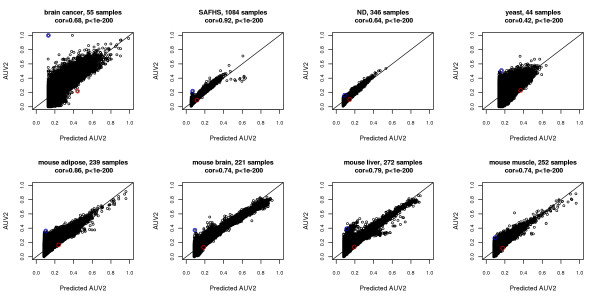
**Comparison of predicted and observed *A***^***MI,UniversalVersion2 ***^**in 8 empirical data sets.** In all data sets, prediction from bicor based on Eq. 18 and observed *A*^*MI*,*UniversalVersion*2 ^are highly correlated (the Pearson correlation and corresponding p-value shown at top). Line y=x is added. Blue and red circles have the same meaning as in Figure 2.

We first calculate bicor and *A*^*MI*,*UniversalVersion*2 ^for all gene pairs in each data set. The two co-expression measures show strong monotonic relationships in most data sets (Figure 2). Then, we predict *A*^*MI*,*UniversalVersion*2^ from bicor based on *F*^*cor*−*MI*^ (Eq. 18). Our predictions are closely related to true *A*^*MI*,*UniversalVersion*2 ^values (Figure 3). These results indicate that most gene pairs satisfy linear relationships in real data applications. Among the 8 data sets, SAFHS shows the strongest association between bicor and *A*^*MI*,*UniversalVersion*2^ (Spearman correlation 0*.*72) and also gives the most accurate *A*^*MI*,*UniversalVersion*2^ prediction (Pearson correlation 0*.*92). A possible reason is that the large samples size (*m *= 1084 ) leads to more accurate estimation of mutual information, thus enhancing the association with bicor and the performance of the prediction function. In contrast, the small sample size (*m *= 44) of the yeast data set adversely affects the calculation of mutual information and hence the prediction performance of *F*^*cor*−*MI*^. In summary, our examples indicate that for most gene pairs, *A*^*MI*,*UniversalVersion*2 ^(Eq. 16) is a monotonic function (cor-MI) of the absolute value of bicor. This finding likely reflects the fact that the vast majority of gene pairs satisfy straight line relationships. This approximation improves with increasing sample size *m*, possibly reflecting more accurate estimation of mutual information.

**Figure 4:**
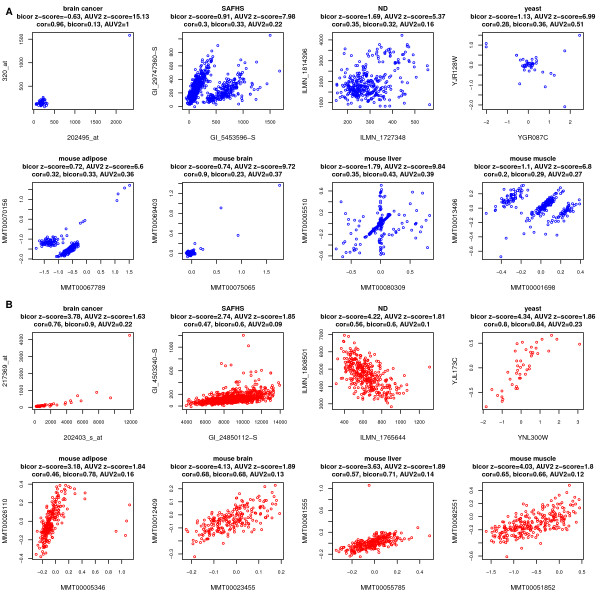
**Gene expression of example probe pairs for which the correlation and mutual information based measures disagree. (A)** Gene expression of probe pairs highlighted by blue circles in Figure 2. **(B)** Gene expression of probe pairs highlighted by red circles in Figure 2. The Pearson correlation, bicor, *A*^*MI*,*UniversalVersion*2 ^values and z-scores of the latter two measures are shown at the top. Mutual information is susceptible to outliers, sometimes detects unusual patterns that are hard to explain, and often misses linear relations that are captured by bicor.

Although *F*^*cor*−*MI*^ reveals a close relationship between bicor and *A*^*MI*,*UniversalVersion*2 ^for most gene pairs, there are cases where the two association measures strongly disagree. In the following, we present scatter plots to visualize the relationships between pairs of genes where MI found a significant relationship while bicor did not and vice versa. To facilitate a comparison between bicor and MI, we standardized each association measure across pairs, which resulted in the Z scores denoted by $\text{Z.M}I_{\text{ij}}=(MI_{\text{ij}}-\text{mean}(\text{MI}))/\sqrt{(}\text{var}(\text{MI}))$ and $\text{Z.bico}r_{\text{ij}}=(\text{bico}r_{\text{ij}}-\text{mean}(\text{bicor}))/\sqrt{(}\text{var}(\text{bicor}))$. Next we selected gene pairs whose value of *Z.MI*_*ij *_was large but *Z.bicor*_*ij *_was low and vice versa. The resulting pairs correspond to the blue and red circles in Figures 2 and 3. To see what dependence patterns drives the discordant behavior of MI and bicor, we used scatter plots to visualize the relationship between the pairs of variables (Figure 4). Gene pairs in Figure (4A) have extreme *A*^*MI*,*UniversalVersion*2^ but insignificant bicor values. Note that the resulting dependencies seem haphazard and may not reflect real biological dependencies. For example, the gene pair in the brain cancer data set exhibits no clear relationships as correctly implied by bicor, while the significant MI value is driven by an array outlier with extremely high expression for both genes. In the SAFHS data, the gene pair exhibits an unusual pattern that is more likely to be the result of batch effects rather than biological signals. The mouse liver data set displays a pairwise pattern that is neither commonly seen nor easily explained. The ND data set shows no obvious patterns at all, making mutual information less trustworthy. On the contrary, gene pairs with significant value of Z.bicor but insignificant *Z.MI *values show approximate linear relationships in all data sets (Figure 4B). Thus, bicor captures gene pairwise relationships more accurately and sensitively than the mutual information based adjacency *A*^*MI*,*UniversalVersion*2^.

In summary, bicor usually detects linear relationships between gene pairs accurately while mutual information is susceptible to outliers, and sometimes identifies pairs that exhibit patterns unlikely to be of biological origin or that exhibit no clear dependency at all. We note that MI results tend to be more meaningful when dealing with a large number of observations (say *m *> 300). Although we only consider 3000 genes with highest variances, our results are highly robust with respect to the number of genes. For example, in Additional file 2, we report results when considering all 23568 genes in the mouse adipose data set or considering 10000 randomly selected genes (rather than with high variance) in the ND data set. These results demonstrate that our findings do not depend on the number of genes.

### Gene ontology enrichment analysis of co-expression modules defined by different networks

Gene co-expression networks typically exhibit modular structure in the sense that genes can be grouped into modules (clusters) comprised of highly interconnected genes (i.e., within-module adjacencies are high). The network modules often have a biological interpretation in the sense that the modules are highly enriched in genes with a common functional annotation (gene ontology categories, cell type markers, etc) [3,30,31]. In this section, we assess association measures (and network construction methods) by the gene ontology (GO) enrichment of their resulting modules in the 8 empirical data sets.

**Figure 5:**
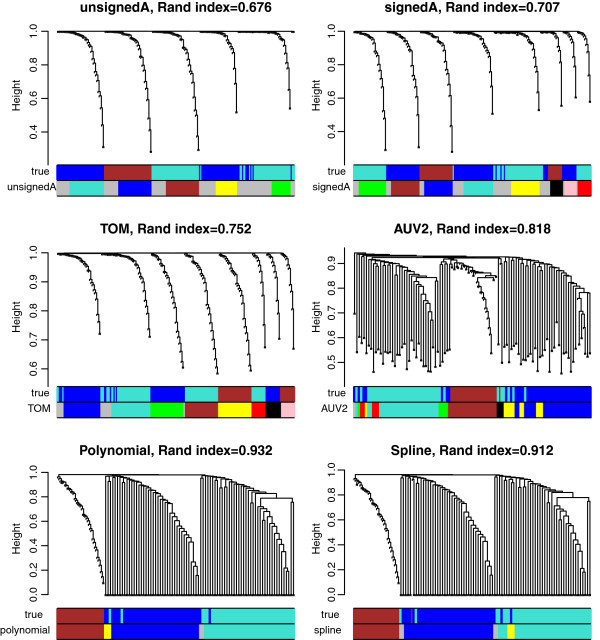
**Module identification based on various network inference methods in simulation with non-linear gene-gene relationships.** The data set is composed of 200 genes across 200 samples. 3 true modules are designed. Two of them, labeled with colors turquoise and blue, contain linear and non-linear (quadratic) gene-gene relationships. For each adjacency, the clustering tree and module colors are shown. True simulated module assignment is shown by the first color band underneath each tree. On top of each panel is the Rand index between inferred and simulated module assignments.

In order to provide an unbiased comparison, we use the same clustering algorithm for module assignment for all networks. Toward this end, we use a module detection approach that has been used in hundreds of publications: modules are defined as branches of the hierarchical tree that results from using 1−*Adjacency* as dissimilarity measure, average linkage, and the dynamic tree cutting method [32]. An example of the module detection approach is illustrated in Figure 5. To provide an unbiased evaluation of GO enrichment of each module, we used the *GOenrichmentAnalysis* R function to test enrichment with respect to all GO terms [33,34] and retained the 5 most significant p-values for each module.

**Figure 6:**
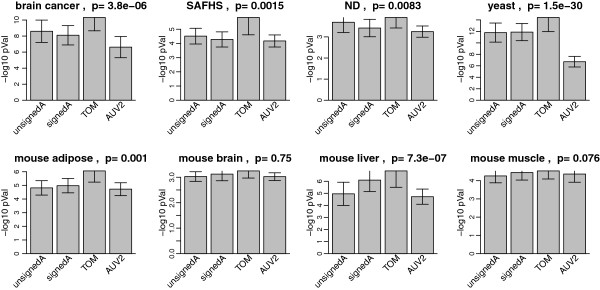
**Gene ontology enrichment analysis comparing *A***^***MI,UniversalVersion2 ***^**with bicor based adjacencies in 8 empirical data sets.** 5 best GO enrichment p-values from all modules identified using each adjacency are log transformed, pooled together and shown as barplots. Error bars stand for 95% confidence intervals. On top of each panel is a p-value based on multi-group comparison test. TOM outperforms the others in all 8 data sets.

**Figure 7:**
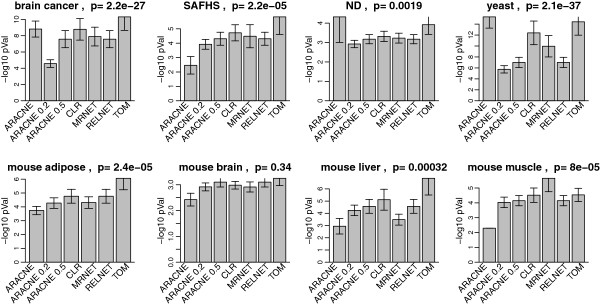
**Gene ontology enrichment analysis comparing TOM with MI based adjacencies in 8 empirical data sets.** 5 best GO enrichment p-values from all modules identified using each adjacency are log transformed, pooled together and shown as barplots. Error bars stand for 95% confidence intervals. On top of each panel is a p-value based on multi-group comparison test. TOM outperforms the others in 5 data sets. ARACNE(*ε *= 0 ) wins in two data sets, making it the second best.

The 10 different adjacencies considered here are described in the last 2 columns of Table 1. We first compare modules based on *A*^*MI*,*UniversalVersion*2 ^with those resulting from 3 bicor based networks: unsigned adjacency (unsignedA, Eq. 29), signed adjacency (signedA, Eq. 28) and Topological Overlap Matrix (TOM, Eq. 30) based on signed adjacency. GO enrichment p-values of modules in the 8 real data applications are summarized as barplots in Figure 6. Figure 6 indicates that, in terms of gene ontology enrichment, TOM is the best bicor based gene co-expression network construction method, and it is superior to *A*^*MI*,*UniversalVersion*2^. Note that signed correlation network coupled with the topological overlap transformation exhibit the most significant GO enrichment p-values in all data sets, and the difference is statistically significant (*p *< 0*.*05) in 6 out of 8 comparisons. The effect of module size is discussed below. An obvious question is whether the performance of MI can be improved when using an alternative MI based network inference method. To address this, we compared the performance of the signed correlation network (with TOM) versus 4 commonly used mutual information: ARACNE, CLR, MRNET and RELNET (described in Materials and Methods). ARACNE allows one to choose a tolerance threshold *ε* ranging from 0 to 1. As *ε*increases, more edges of the ARACNE network will be preserved. We evaluated ARACNE (*ε *= 0), ARACNE (*ε *= 0*.*2) and ARACNE (*ε *= 0*.*5 ) into our comparison. Similarly to Figures 6 and 7 summarizes the GO enrichment p-values of modules in the 8 real data applications. TOM leads to the highest enrichment p-values in 5 cases, and the difference is statistically significant in 4 of them. In two applications, ARACNE (*ε *= 0) performs best, and MRNET performs best in one application. We need to point out that another mutual information based method, maximal information coefficient (MIC) [35], has been proposed recently. Although computational intensive, the MIC has clear theoretical advantages when it comes to capturing general dependence patterns. Additional file 3 compares the performance of MIC with that of TOM when it comes to GO ontology enrichment. TOM clearly outperforms MIC to identify GO enriched modules in 6 out of 7 data sets which may suggest that MIC tends to overfit the data in these applications. SAFHS data set is not included because the computation of MIC was time-consuming on this large data set.

**Table 1:** Types of networks and characteristics

**Network type**	**Used here**	**Examples**	**Variable**	**Ease of estimation**	**Utility for modeling**						**Adjacencies**	**Used in GO**
			**types**								**discussed**	**enrichment**
											**this article**	**analysis**
					**GRN**	**Reduce**	**Direct**	**Time**	**Nonlin.**	**Sign**		
**Correlation network**	Yes	WGCNA [5]	Numeric	Easy	Yes	Yes	No	Maybe	No	Yes	unsignedA	Yes
											signedA	Yes
											TOM	Yes
**Polynomial or**	Yes	WGCNA [5]	Numeric	Moderate	Yes	Yes	No	Maybe	Yes	No	*polyR*^2^	No
**Spline regression**											*splineR*^2^	No
**network**												
**Mutual information network**	Yes	ARACNE	Discretized	Moderate	Yes	Not clear	No	Maybe	Yes	No	ASU	No
		[9], RELNET	numeric,								AUV1	No
		[6,28], CLR	categorical								AUV2	Yes
		[26], MRNET									ARACNE	Yes
		[27], MIC [35]									ARACNE0.2	Yes
											ARACNE0.5	Yes
											CLR	Yes
											MRNET	Yes
											RELNET	Yes
											MIC	Yes
**Boolean network**	No	Boolean network [71]	Dichoto-mized numeric	Moderate	Yes	Not clear	Yes	Yes	NA	NA	No	No
**Probabilistic network**	No	Bayesian network [72,73]	Any	Hard	Yes	Not clear	Yes	Yes	Yes	Yes	No	No

For each network method, the table reports what kinds of biological insights can be gained and what kind of data can be analyzed. Column “GRN” indicates whether the network has been (or can be) used for studying gene regulatory networks. Column “Reduce” indicates whether the method has been used for reducing high dimensional data (e.g. via modules and their representatives). Column “Direct” indicates whether the the network can encode directional information. Column “time” indicates whether the network method is suited for studying time series data. Column “Nonlin. ” indicates whether the network can capture non-linear relationships between pairs of variables (represented as nodes). Column “Sign” indicates whether the network adjacency provides information on the sign of the relationship between two variables, e.g. a correlation coefficient can take on positive and negative values. The table entry “NA” stands for not applicable. Adjacencies discussed in this article: unsignedA: unsigned bicor; signedA: signed bicor; TOM: TOM transformed signed bicor; ASU: *A*^*MI*,*SymmetricUncertainty*^; AUV1: *A*^*MI*,*UniversalVersion*1^; AUV2: *A*^*MI*,*UniversalVersion*2^; ARACNE: ARACNE, *ε *= 0 ; ARACNE0.2: ARACNE, *ε *= 0*.*2 ; ARACNE0.5: ARACNE, *ε *= 0*.*5.

**Overall, these unbiased comparisons show that signed correlation networks coupled with the topological overlap transformation outperform the commonly used mutual information based algorithms when it comes to GO enrichment of modules**.

### Polynomial and spline regression models as alternatives to mutual information

A widely noted advantage of mutual information is that it can detect general, possibly non-linear, dependence relationships. However, estimation of mutual information poses multiple challenges ranging from computational complexity to dependency on parameters and difficulties with small sample sizes. Standard polynomial and spline regression models can also detect non-linear relationships between variables. While perhaps less general than MI, relatively simple polynomial and spline regression models avoid many of the challenges of estimating MI while adequately modeling a broad range of non-linear relationships. In addition to being computationally simpler and faster, regression models also make available standard statistical tests and model fitting indices. Thus, in this section we examine polynomial and spline regression as alternatives to MI for capturing non-linear relationships between gene expression profiles. We define association measures based on polynomial and spline regression models and study their performance.

#### Networks based on polynomial and spline regression models

Consider two random variables *x* and *y* and the following polynomial regression model of degree 3:

(22) $$E(y|x)=\beta_{0}+\beta_{1}x+\beta_{2}x^{2}+\beta_{3}x^{3}.$$

The model fitting index *R*^2^(*x*,*y*) (described in Materials and Methods) can be used to evaluate the fit of the model. One can then reverse the roles of *x* and *y* to arrive at a model fitting index *R*^2^(*y*,*x*) . In general, *R*^2^(*x*,*y*) ≠* R*^2^(*y*,*x*).

Now consider a set of *n* variables *x*_1_,…,*x*_*n*_. One can then calculate pairwise model fitting indices $\left(R_{\text{ij}}^{2}=R^{2}(x_{i},x_{j})\right)$ which can be interpreted as the elements of an *n *×* n* association matrix $\left(\left(R_{\text{ij}}^{2}\right)\right)$. This matrix is in general non-symmetric and takes on values in [0,1] , with diagonal values equal to 1. A large value indicates a close relationship between variables *x*_*i*_ and *x*_*j*_. To define an adjacency matrix, we symmetrize $\left(\left(R_{\text{ij}}^{2}\right)\right)$ through Eqs. 3, 4 or 5.

Spline regression models are also known as local polynomial regression models [36]. Local refers to the fact that these models amount to fitting models on subintervals of the range of *x*. The boundaries of subintervals are referred to as knots. In analogy to polynomial models, we build natural cubic spline model for all pairs of *x*_*i*_,*x*_*j*_. We use the following rule of thumb for the number of knots: if *m *> 100 use 5 knots, if *m *< 30 use 3 knots, otherwise use 4 knots. We then calculate model fitting indices and create corresponding network adjacencies. (Details of spline model construction can be found in Materials and Methods.)

Compared to spline regression, polynomial regression models have a potential shortcoming: the model fit can be adversely affected by outlying observations. A single outlying observation (*x*_*u*_,*y*_*u*_) can “bend” the fitting curve into the wrong direction, i.e. adversely affect the estimates of the *β *coefficients. Spline regression alleviates this problem by fitting model on sub-intervals of the range of *x*.

**Figure 8:**
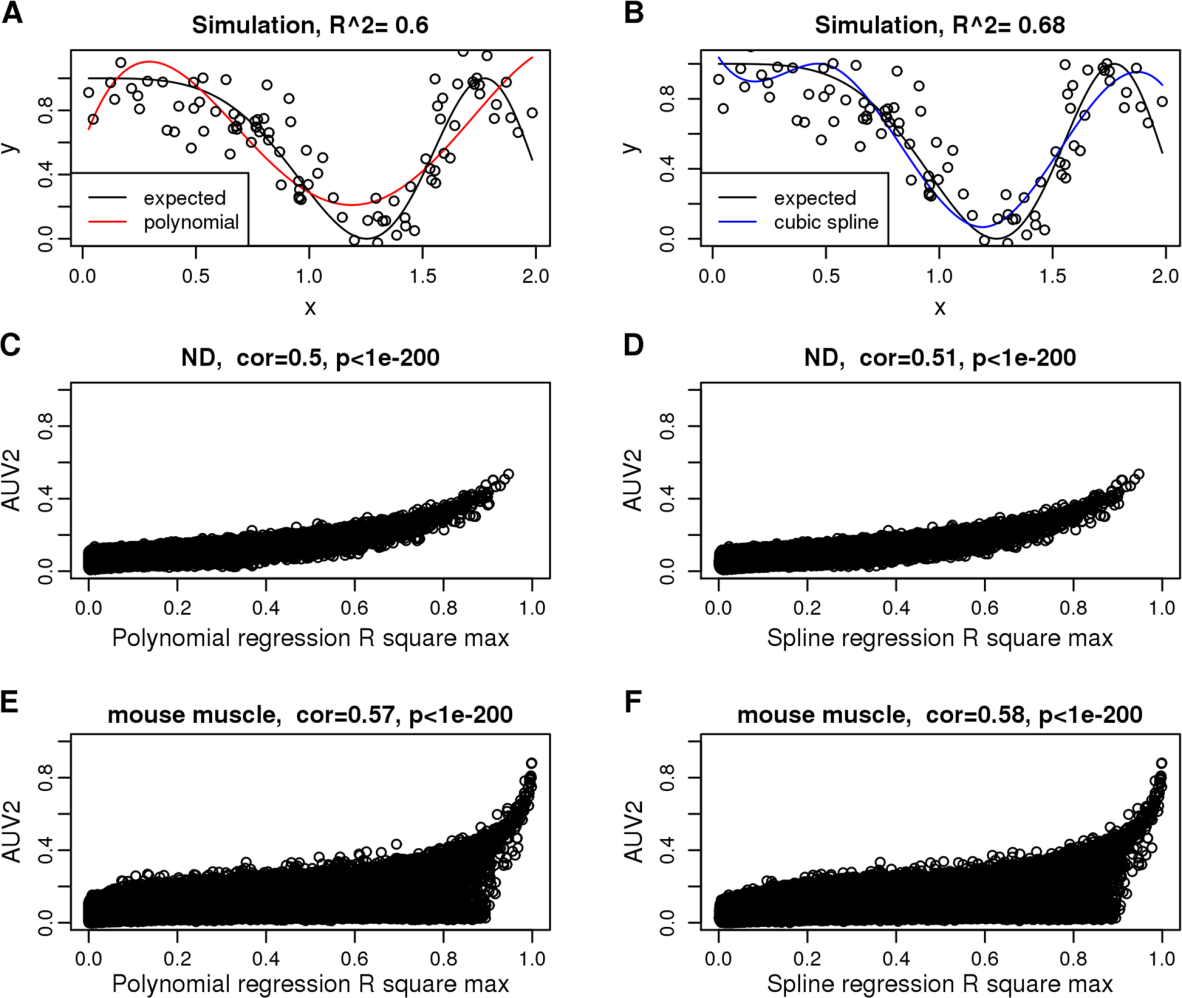
**Fitting polynomial and spline regression models to measure non-linear relationships. (A-B)** A pair of simulated data *x*,*y*(black dots) with the black curve illustrating the true expected value *E*(*y*) given x, where *E*(*y*|*x*) =* cos*(*x*^2^)^2^. The red curve shows the fit of a polynomial regression model with degree *d *= 4 . The blue curve shows the fit of a cubic spline regression model with 2 knots. Fitting indices of the two models are shown at the top. In simulation, polynomial and cubic spline regression models can properly discover non-linear relations. **(C-D)** Comparisons of regression models and mutual information based co-expression measures in the ND data set. Co-expression of probe pairs is measured with polynomial (d = 3)/cubic spline regressions (x-axis) and mutual information *A*_*MI*,*UniversalVersion*2_(y-axis). The Spearman correlation and p-value of the two measures are shown at the top. **(E-F)** Comparisons in the mouse muscle data set. *A*_*MI*,*UniversalVersion*2 _has a stronger correlation with regression models than with bicor, indicating that the first two measures can capture certain common non-linear patterns.

Figure 8 (A-B) illustrates the use of regression models for measuring non-linear relationships. In simulation, polynomial and cubic spline regression can correctly capture non-linear trends.

#### Relationship between regression and MI based networks

Previously, we discussed the relationship between correlation and mutual information based adjacencies in simulations where *x* and *y *represent samples from a bivariate normal distribution. Here, we consider the performance of polynomial and spline association measure in the same scenario (Additional file 4). With all *x*,*y* pairs following linear relationships, both regression models reduce to simple linear models, and perform almost identically to correlation based measures (panel (A) and (C)). We find that the cor-MI function introduced previously also allows us to relate spline and polynomial regression based networks to the MI based network (panel (B) and (D)), e.g. $\text{AUV}2_{\text{ij}}\approx F^{\text{cor}-\text{MI}}(\sqrt{\text{max}(R^{2}(x_{i},x_{j}),R^{2}(x_{j},x_{i}))})$. Note that different symmetrization methods (Eq. 3) applied *R*^2 ^result in similar adjacencies in our applications (refer to Additional file 5), thus it’s valid to use any of them.

In addition, our empirical data show that regression models and mutual information adjacency *A*^*MI*,*UniversalVersion*2 ^are highly correlated, and the relationship is stronger than that between bicor and *A*^*MI*,*UniversalVersion*2^ (Figure 8 C-F). This indicates that *A*^*MI*,*UniversalVersion*2 ^and regression models discover some common gene pairwise non-linear relations that can not be identified by correlations. The Neurological Disease (ND) and mouse muscle sets are shown in Figure 8 as representatives. A detailed analysis of all data sets can be found in Additional file 5.

#### Simulations for module identification in data with non-linear relationships

**Figure 9:**
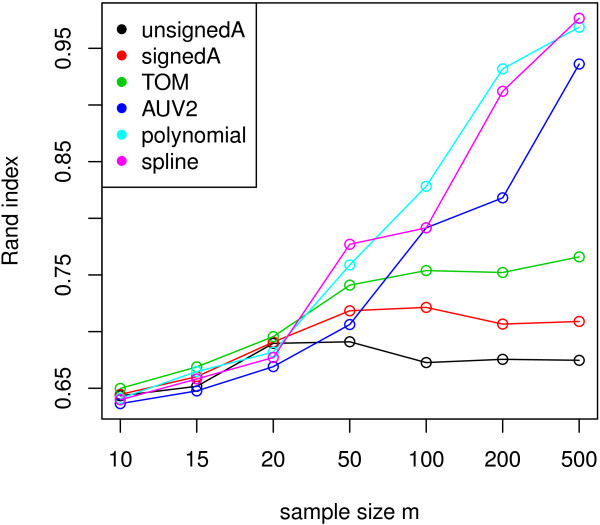
**Rand indices in simulations with various number of observations.** Simulation sample size versus Rand indices between inferred and simulated module assignments from different network inference methods. increase as the simulation data set contains more samples. Non-linear measures, especially polynomial and spline regression models, outperform other measures as sample size increases.

Our empirical studies show that most gene pairs satisfy linear relationships, which implies that correlation based network methods perform well in practice. But one can of course simulate data where non-linear association measures (such as MI, spline *R*^2^) outperform correlation measures when it comes to module detection. To illustrate this point, we simulated data with non-linear gene-gene relationships. Here we simulated 200 genes in 3 network modules across 200 samples. Two of the simulated modules, labeled for convenience by the colors turquoise and blue, contain linear and non-linear (quadratic) gene-gene relationships (Figure 5). We then use several different network inference methods to construct networks and define modules. To evaluate how well each network inference method recovers the simulated modules, we use the Rand index between the inferred and simulated module assignment. In this case, non-linear association measures, i.e. AUV2, polynomial and spline regression, identify modules more accurately than correlation based measures (Figure 5). In networks based on correlations, the simulated turquoise and blue modules are clearly divided into two separate ones, indicating that they miss the non-linear relationships within these two modules. In contrast, regression models capture non-linear gene pairwise relations and correctly assign these genes into the same modules. To study the effect of the number of observations, we repeated the analysis for *m* ranging from 10 to 500. Figure 9 shows that non-linear association measures, especially regression models, outperform correlation based measures as data sample size increases. Note that polynomial and spline regression based co-expression measures perform as well as MI based networks in this situation. Overall, our results validate the usage of polynomial and spline regression models as alternatives to mutual information for detecting non-linear relationships.

### Overview of network methods and alternatives

A thorough review of network methods is beyond our scope and we point the reader to the many many review articles [37-40]. But Table 1 describes not only the methods used in this article but also alternative approaches. Table 1 also describes the kind of biological insights that can be gained from these network methods. As a rule, association networks (based on correlation or MI) are ill suited for causal analysis and for encoding directional information. While association networks such as WGCNA or ARACNE have been been successfully used for gene regulatory networks (GRNs) [13], a host of alternatives are available. For example, the DREAM (Dialogue for Reverse Engineering Assessments and Methods) project has repeatedly tackled this problem [41-43]. A limitation of our study is that we are focusing on undirected (as opposed to directed, causal models). Structural equation models, Bayesian networks, and other probabilistic graphical models are widely used for studying causal relationships. Many authors have proposed to use Bayesian networks for analyzing gene expression data [44-47] and for generating causal networks from observational data [48] or genetic data [49,50].

While it is beyond our scope to evaluate network inference methods for time series data (reviewed in [51]), we briefly mention several approaches. A (probabilistic) Boolean network [52] is a special case of a discrete state space model that characterizes a system using dichotomized data. A Bayesian network is a graph-based model of joint multivariate probability distributions that captures properties of conditional independence between variables [45]. Such models are attractive for their ability to describe complex stochastic processes and for modeling causal relationships. Several articles describe the relationship between Boolean networks and dynamic Bayesian networks when it comes to models of gene regulatory relationships [47,53]. Finally, we mention that correlation network methodology can be adapted to model time series data, e.g. many authors have proposed to use a time-lagged correlation measure for inferring gene regulatory networks [54].

A large part of GRN research focuses on the accurate assessment of individual network edges, e.g. [55-58] so many of these methods are not designed as data reduction methods. In contrast, correlation network methods, such as WGCNA, are highly effective at reducing high dimensional genomic data since modules can be represented by their first singular vector (referred to as module eigengene) [21,59].

## Discussion

This article presents the following theoretical and methodological results: i) it reviews the relationship between the MI and a likelihood ratio test statistic in case of two categorical variables, ii) it presents a novel empirical formula for relating correlation to MI when the two variables satisfy a linear relationship, and iii) it describes how to use polynomial and spline regression models for defining pairwise co-expression measures that can detect non-linear relationships.

Mutual information has several appealing information theoretic properties. A widely recognized advantage of mutual information over correlation is that it allows one to detect non-linear relationships. This can be attractive in particular when dealing with time series data [60]. But mutual information is not unique in being able to detect non-linear relationships. Standard regression models such as polynomial and spline models can also capture non-linear relationships. An advantage of these models is that well established likelihood based statistical estimation and testing procedures are available. Regression models allow one to calculate model fitting indices that can be used to define network adjacencies as well as flag possible outlying observations by analyzing residuals.

For categorical variables, mutual information is (asymptotically) equivalent to other widely used statistical association measures such as the likelihood ratio statistic or the Pearson chi-square test. In this case, all of these measures (including MI) are arguably optimal association measures. Interpreting MI as a likelihood ratio test statistic facilitates a straightforward approach for adjusting the association measure for additional covariates.

We and others [14] have found close relationships between mutual information and correlation based co-expression networks. Our comprehensive empirical studies show that mutual information is often highly related to the absolute value of the correlation coefficient. We observe that when robust correlation and mutual information disagree, the robust correlation findings appear to be more plausible statistically and biologically. We found that network modules defined using robust correlation exhibit on average higher enrichment in GO categories than modules defined using mutual information. Since our empirical studies involved expression data measured on a variety of platforms and normalized in different ways, we expect that our findings are broadly applicable.

The correlation coefficient is an attractive alternative to the MI for the following reasons. First, the correlation can be accurately estimated with relatively few observations and it does not require the estimation of the (joint) frequency distribution. Estimating the joint density needed for calculating MI typically requires larger sample sizes. Second, the correlation does not depend on hidden parameter choices. In contrast, MI estimation methods involve (hidden) parameter choices, e.g. the number of bins when a discretization method is being used. Third, the correlation allows one to quickly calculate p-values and false discovery rates since asymptotic tests are available (Additional file 1). In contrast, it is computationally challenging to calculate a permutation test p-value for the mutual information between two discretized vectors. Fourth, the sign of the correlation allows one to distinguish positive from negative relationships. Signed correlation networks have been found useful in biological applications [22] and our results show that the resulting modules tend to be more significantly enriched with GO terms that those of networks that ignore the sign information. Fifth, modules comprised of highly correlated vectors can be effectively summarized by the module eigennode (the first principal component of scaled vectors). Sixth, the correlation allows for a straightforward angular interpretation, which facilitates a geometric interpretation of network methods and concepts [59]. For example, intramodular connectivity can be interpreted as module eigennode based connectivity.

Our empirical studies show that a signed weighted correlation network transformed via the topological overlap matrix transformation often leads to the most significant functional enrichment of modules. The recently developed maximal information coefficient [35] has clear theoretical advantages when it comes to measuring general dependence patterns between variables but our results show that the biweight midcorrelation coupled with the topological overlap measure outperforms the MIC when it comes to the GO ontology enrichment of resulting coexpression modules.

While defining mutual information for categorical variables is relatively straightforward, no consensus seems to exist in the literature on how to define mutual information for continuous variables. A major limitation of our study is that we only studied MI measures based on discretized continuous variables. For example, the cor-MI function for relating correlation to MI only applies when an equal width discretization method is used with $\text{no.bins}=\sqrt{m}$.

A second limitation concerns our gene ontology analysis of modules identified in networks based on various association measures in which we found that the correlation based topological overlap measure (TOM) leads to co-expression modules that are more highly enriched with GO terms than those of alternative approaches. A potential problem with our approach is that the enrichment p-values often strongly depend on (increase with) module sizes, and TOM tends to lead to larger modules. To address this concern, in Additional file 6 we show the enrichment p-values as a function of module size for modules identified by TOM and by AUV2. It turns out that in most studies, the enrichment of modules defined by TOM is better than that of comparably sized modules defined by AUV2.

A third limitation concerns our use of the bicor correlation measure as opposed to alternatives (e.g. Pearson or Spearman correlation). In our study we find that all 3 correlation measures lead to very similar findings (Additional file 7).

## Conclusions

Our simulation and empirical studies suggest that mutual information can safely be replaced by linear regression based association measures (e.g. bicor) in case of stationary gene expression measures (which are represented by quantitative variables). To capture general monotonic relationships between such variables, one can use the Spearman correlation. To capture more complicated dependencies, one can use symmetrized model fitting statistics from a polynomial or spline regression model. Regression based association measures have the advantage of allowing one to include covariates (conditioning variables). In case of categorical variables, mutual information is an appropriate choice since it is equivalent to an association measure (likelihood ratio test statistic) of a generalized linear regression model but categorical variables rarely occur in the context of modeling relationships between gene products.

## Materials and Methods

### Empirical gene expression data sets description

**Brain cancer data set.** This data set was composed of 55 microarray samples of glioblastoma (brain cancer) patients. Gene expression profiling were performed with Affymetrix high-density oligonucleotide microarrays. A detailed description can be found in [61].

**SAFHS data set.** This data set [62] was derived from blood lymphocytes of randomly ascertained participants enrolled independent of phenotype in the San Antonio Family Heart Study. Gene expression profiles of 1084 samples were measured by Illumina Sentrix Human Whole Genome (WG-6) Series I BeadChips.

**ND data set.** This blood lymphocyte data set consisted of 346 samples from patients with neurological diseases. Illumina HumanRef-8 v3.0 Expression BeadChip were used to measure their gene expression profiles.

**Yeast data set.** The yeast microarray data set was composed of 44 samples from the Saccharomyces Genome Database (http://db.yeastgenome.org/cgi-bin/SGD/expression/expressionConnection.pl). Original experiments were designed to study the cell cycle [63]. A detailed description of the data set can be found in [64].

**Tissue-specific mouse data sets.** This study uses 4 tissue-specific gene expression data from a large *F*_2_ mouse intercross (B × H) previously described in [65,66]. Specifically, the surveyed tissues include adipose (239 samples), whole brain (221 samples), liver (272 samples) and muscle (252 samples).

### Definition of Biweight Midcorrelation

Biweight midcorrelation (bicor) is considered to be a good alternative to Pearson correlation since it is more robust to outliers [67]. In order to define the biweight midcorrelation of two numeric vectors *x *= (*x*_1_,…,*x*_*m*_) and *y *= (*y*_1_,…,*y*_*m*_), one first defines *u*_*i*_*v*_*i *_with *i *= 1,…,*m*:

(23) $$\begin{array}{c} u_{i}=\frac{x_{i}-\text{med}(x)}{9\text{mad}(x)}\\ v_{i}=\frac{y_{i}-\text{med}(y)}{9\text{mad}(y)} \end{array}$$

where *med*(*x*) is the median of *x*, and *mad*(*x*) is the median absolute deviation of *x*. This leads us to the definition of weight *w*_*i *_for *x*_*i*_, which is,

(24) $$w_{i}^{\left(x\right)}={\left(1-u_{i}^{2}\right)}^{2}I(1-|u_{i}|)$$

where the indicator *I*(1−|*u*_*i*_|) takes on value 1 if 1−|*u*_*i*_| > 0 and 0 otherwise. Therefore, $\left(w_{i}^{\left(x\right)}\right)$ ranges from 0 to 1. It decreases as *x*_*i *_gets away from *med*(*x*), and stays at 0 when *x*_*i *_differs from *med*(*x*) by more than 9*mad*(*x*). An analogous weight $\left(w_{i}^{\left(y\right)}\right)$ can be defined for *y*_*i*_. Given the weights, we can define biweight midcorrelation of *x* and *y* as:

(25) $$\text{bicor}(x,y)=\frac{\overset{m}{\underset{i=1}{\sum }}(x_{i}-\text{med}(x))w_{i}^{\left(x\right)}(y_{i}-\text{med}(y))w_{i}^{\left(y\right)}}{\sqrt{\overset{m}{\underset{j=1}{\sum }}{\left[(x_{j}-\text{med}(x))w_{j}^{\left(x\right)}\right]}^{2}}\sqrt{\overset{m}{\underset{k=1}{\sum }}{\left[(y_{k}-\text{med}(y))w_{k}^{\left(y\right)}\right]}^{2}}}.$$

A modified version of biweight midcorrelation is implemented as function *bicor* in the WGCNA R package [5,20]. One major argument of the function is “maxPOutliers”, which caps the maximum proportion of outliers with weight *w*_*i *_= 0. Practically, we find that *maxPOutliers *= 0*.*02 detects outliers efficiently while preserving most data. Therefore, 0*.*02 is the value we utilize in this study.

### Types of correlation based gene co-expression networks

Given the expression profile *x*,the co-expression similarity *s*_*ij *_between genes *i* and *j* can be defined as:

$$s_{\text{ij}}=|\text{cor}(x_{i},x_{j})|.$$

An **unweighted network adjacency*** A*_*ij *_between gene expression profiles ***x***_*i*_ and ***x***_*j*_ can be defined by hard thresholding the co-expression similarity *s*_*ij *_as follows

(26) $$A_{\text{ij}}=\left\{\begin{array}{ll} 1 & \text{if}\;s_{\text{ij}}\geq \tau \\ 0 & \text{otherwise,} \end{array}\right)$$

where *τ* is the ‘hard’ threshold parameter. Hard thresholding of the correlation leads to simple network concepts (e.g., the gene connectivity equals the number of direct neighbors) but it may lead to a loss of information.

To preserve the continuous nature of the co-expression information, we define the **weighted network adjacency** between 2 genes as a power of the absolute value of the correlation coefficient [4,61]:

(27) $$A_{\text{ij}}=s_{\text{ij}}^{\beta },$$

with *β *≥ 1. This soft thresholding approach emphasizes strong correlations, punishes weak correlations, and leads to a weighted gene co-expression network.

An important choice in the construction of a correlation network concerns the treatment of strong negative correlations. In **signed networks** negatively correlated nodes are considered unconnected. In contrast, in **unsigned networks** nodes with high negative correlations are considered connected (with the same strength as nodes with high positive correlations). As detailed in [4,22], a signed weighted adjacency matrix can be defined as follows

(28) $$A_{\text{ij}}={\left(0.5+0.5\text{cor}(x_{i},x_{j})\right)}^{\beta }$$

and an unsigned adjacency by

(29) $$A_{\text{ij}}=|\text{cor}(x_{i},x_{j}){|}^{\beta }\;.$$

*β* is default to 6 for unsigned adjacency and 12 for signed adjacency. The choice of signed vs. unsigned networks depends on the application; both signed [22] and unsigned [30,61,65] weighted gene networks have been successfully used in gene expression analysis.

### Adjacency function based on topological overlap

The topological overlap matrix (TOM) based adjacency function *A*_*TOM*_ maps an original adjacency matrix *A*^*original *^to the corresponding topological overlap matrix, i.e.

(30) $$\begin{array}{c} A_{\text{TOM}}{\left(A^{\text{original}}\right)}_{\text{ij}}\;=\;\frac{\underset{l\neq i,j}{\sum }A_{\text{il}}^{\text{original}}A_{l,j}^{\text{original}}\;+\;A_{\text{ij}}^{\text{original}}}{\text{min}(\underset{l\neq i}{\sum }A_{\text{il}}^{\text{original}},\underset{l\neq j}{\sum }A_{\text{jl}}^{\text{original}})\;-\;A_{\text{ij}}^{\text{original}}\;+\;1}. \end{array}$$

The TOM based adjacency function *A*_*TOM*_ is particularly useful when the entries of *A*^*original *^are sparse (many zeroes) or susceptible to noise. This replaces the original adjacencies by a measure of interconnected that is based on shared neighbors. The topological overlap measure can serve as a filter that decreases the effect of spurious or weak connections and it can lead to more robust networks [17,18,68].

### Mutual-information based network inference methods

There are 4 commonly used mutual-information based network inference methods: RELNET, CLR, MRNET and ARACNE. In order to identify pairwise interactions between numeric variables *x*_*i*_,*x*_*j*_, all methods start by estimating mutual information *MI*(*x*_*i*_,*x*_*j*_).

#### RELNET

The relevance network (RELNET) approach [6,28] thresholds the pairwise measures of mutual information by a threshold *τ*. However, this method suffers from a significant limitation that vectors separated by one or more intermediaries (indirect relationships) may have high mutual information without implying a direct interaction.

#### CLR

The CLR algorithm [26] is based on the empirical distribution of MI. It first defines a score *z*_*i*_ given the mutual information *MI*(*x*_*i*_,*x*_*j*_) and the sample mean *μ*_*i*_ and standard deviation *σ*_*i*_ of the empirical distribution of mutual information *MI*(*x*_*i*_,*x*_*k*_),*k *= 1,…,*n*:

(31) $$z_{i}=\max\left(0,\frac{\text{MI}(x_{i},x_{j})-\mu_{i}}{\sigma_{i}}\right).$$

*z*_*j*_ can be defined analogously. In terms of *z*_*i*_,*z*_*j*_, the score used in CLR algorithm can be expressed as $\left(z_{\text{ij}}=\sqrt{z_{i}^{2}+z_{j}^{2}}\right)$.

#### MRNET

MRNET [27] infers a network by repeating the maximum relevance/minimum redundancy (MRMR) feature selection method for all variables. The MRMR method starts by selecting the variable *x*_*i*_ having the highest mutual information with target y. Next, given a set *S* of selected variables, the criterion updates *S* by choosing the variable *x*_*k*_ that maximizes *u*_*j*_−*r*_*j*_ where *u*_*j*_ is a relevance term and *r*_*j*_ is a redundancy term. In particular,

(32) $$\begin{array}{l} u_{j}=\text{MI}(x_{k},y) \end{array}$$

(33) $$\begin{array}{l} r_{j}=\frac{1}{\left|S\right|}\underset{x_{i}\in S}{\sum }\text{MI}(x_{k},x_{i}) \end{array}$$

The score of each pair *x*_*i*_ and *x*_*j*_ will be the maximum score of the one computed when *x*_*i*_ is the target and the one computed when *x*_*j *_is the target.

#### ARACNE

The ARACNE [9] (Algorithm for the Reconstruction of Accurate Cellular Networks) developed by Andrea Califano’s group is an extension of RELNET. Given the limitation of RELNET, ARACNE removes the vast majority of indirect candidate interactions using a well-known information theoretic property, the **data processing inequality** (DPI). The DPI applied to association networks states that if variables *x*_*i*_ and *x*_*j*_ interact only through a third variable *x*_*k*_, then

(34) $$\text{MI}(x_{i},x_{j})\leq \text{min}(\text{MI}(x_{i},x_{k}),\text{MI}(x_{k},x_{j}))$$

ARACNE starts with a network graph where each pair of nodes with *MI*_*ij *_>* τ *is connected by an edge. The weakest edge of each triplet, e.g. the edge between *i* and *j*, is interpreted as an indirect interaction and is removed if the difference between *min*(*MI*(*x*_*i*_,*x*_*k*_),*MI*(*x*_*k*_,*x*_*j*_)) and *MI*(*x*_*i*_,*x*_*j*_) lies above a threshold *ε*, i.e. the edge is removed if

(35) $$\text{MI}(x_{i},x_{j})\leq \text{min}(\text{MI}(x_{i},x_{k}),\text{MI}(x_{k},x_{j}))-\mathrm{\varepsilon .}$$

The tolerance threshold *ε *could be chosen to reflect the variance of the MI estimator and should decrease with increasing sample size *m*. Using a non-zero tolerance *ε *> 0 can lead to the persistence of some 3-vector loops.

The outputs from RELNET, CLR, MRNET or ARACNE are association matrices. They can be transformed into corresponding adjacencies based on the algorithm discussed in Introduction.

#### MIC

Another mutual information based method is the recently proposed the maximal information coefficient (MIC) [35]. The MIC is a type of maximal information-based nonparametric exploration (MINE) statistics [35]. In our empirical evaluations, we calculate the MIC using the *minerva* R package [69].

### Fitting indices of polynomial regression models

While networks based on the Pearson correlation can only capture linear co-expression patterns there is clear evidence for non-linear co-expression relationships in transcriptional regulatory networks [70]. The following classical regression based approaches can be used for studying non-linear relationships. The polynomial regression model:

(36) $$\begin{array}{l} E(y)=\beta_{0}1+\beta_{1}x+\beta_{2}x^{2}\ldots +\beta_{d}x^{d}\\ \;\;=M\beta \;, \end{array}$$

where

(37) $$M=[1,x,\ldots ,x^{d}]\;.$$

One can show that the least squares estimate of the parameter vector $\hat{\beta }$ is

$$\hat{\beta }={\left(M^{\tau }M\right)}^{-}M^{\tau }\;y,$$

where^-^ denotes the (pseudo) inverse, and ^*τ *^denotes the transpose of a matrix.

Given $\hat{\beta }$, we can calculate the fitting index *R*^2 ^as:

(38) $$R^{2}=\text{cor}{\left(y,ŷ\right)}^{2}=\text{cor}{\left(y,M\hat{\beta }\right)}^{2}$$

In the context of a regression model, *R*^2 ^is also known as the proportion of variation of y explained by the model.

### Spline regression model construction

To investigate the relationship between variable *x* and *y*, one can use another textbook method from the arsenal of statisticians: spline regression models. Here knots are used to decide boundaries of the sub-intervals. They are typically pre-specified, e.g. based on quantiles of *x*. The choice of the knots will affect the model fit. It turns out that the values of the knots (i.e. their placement) is not as important as the number of knots. We use the following rule of thumb for the number of knots: if *m *> 100 use 5 knots, if *m *< 30 use 3 knots, otherwise use 4 knots.

To ensure that fit between *y* and *x* satisfies a continuous relationship, we review the **hockey stick function**()_ + _ to transform *x* :

(39) $${\left(s\right)}_{+}=\left\{\begin{array}{ll} s & \text{if}\;\text{s}\geq 0\\ 0 & \text{if}\;\text{s}<0. \end{array}\right)$$

This function can also be applied to the components of a vector, e.g. (*x*)_ + _denotes a vector whose negative components have been set to zero. So (*x*−*knot*1)_ + _ is a vector whose u-th component equals *x*[*u*]−*knot*1 if *x*[*u*]−*knot*1 ≥ 0 and 0 otherwise.

We are now ready to describe **cubic spline regression model**, which fits polynomial of degree 3 to sub-intervals. The general form of a cubic spline with 2 knots is as follows

(40) $$\begin{array}{l} E(y)=\beta_{0}1+\beta_{1}x+\beta_{2}x^{2}+\beta_{3}x^{3}+\beta_{4}{\left(x-\text{kno}t_{1}\right)}_{+}^{3}\\ \;+\beta_{5}{\left(x-\text{kno}t_{2}\right)}_{+}^{3}. \end{array}$$

The knot parameters (numbers) *knot*_1_,*knot*_2_,… are chosen before estimating the parameter values. Analogous to polynomial regression, *R*^2^ can be calculated as the association measure between *x* and *y*. This method guarantees the smoothness of the regression line and restrict the influence of each observation to its local sub-interval.

### Other networks

Boolean network [71] and Probabilistic network [72,73] are briefly mentioned in Table 1.

### Availability of software

**Project name:** Adjacency matrix for non-linear relationships

 Project home page: http://www.genetics.ucla.edu/labs/horvath/CoexpressionNetwork/Rpackages/WGCNA

**Operating system(s):** Platform independent

**Programming language:** R

**Licence:** GNU GPL 3

The following functions described in this article have been implemented in the WGCNA R package [5]. Function *adjacency.polyReg* and *adjacency.splineReg* calculate polynomial and spline regression *R*^2^ based adjacencies. Users can specify the *R*^2 ^symmetrization method. Function *mutualInfoAdjacency* calculates the mutual information based adjacencies *A*^*MI*,*SymmetricUncertainty*^ (Eq. 14), *A*^*MI*,*UniversalVersion*1^ (Eq. 15) and *A*^*MI*,*UniversalVersion*2^ (Eq. 16). Function *AFcorMI* implements the *F*^*cor*−*MI*^prediction function 18 for relating correlation with mutual information.

## Abbreviations

MI: Mutual information; Bicor: Biweight midcorrelation; MIC: Maximal information coefficient; ARACNE: Algorithm for the reconstruction of accurate cellular networks; GO: Gene ontology; LRT: Likelihoood ratio test; TOM: Topological overlap matrix; WGCNA: Weighted correlation network analysis.

## Competing interests

We declare no conflict of interest.

## Authors’ contributions

LS and SH performed the research; LS, SH, and PL wrote the paper and developed R software functions. SH designed the research. All authors read and approved the final manuscript.

## Supplementary Material

Additional file 1 **Detailed methods descriptions.** In this document, we provide detail information of entropy, mutual information, likelihood ratio test statistics and p-value calculation of correlation coefficients. Click here for file

Additional file 2 **Empirical analysis using large number of genes in the mouse adipose and ND data sets.** Page one is an empirical analysis using all 23568 genes without restricting to 3000 genes for the mouse adipose data set. (A) Absolute value of bicor versus *A*^*MI*,*UniversalVersion*2^. One million randomly sampled gene pairs are plotted to reduce computational burden. The two measures show good monotonic relationship. The red curve predicts *A*^*MI*,*UniversalVersion*2^ from bicor. The blue circle highlights the probe pair with the highest *A*^*MI*,*UniversalVersion*2^ z-score among those with insignificant bicor z-scores (less than 1*.*9 ); the red circle highlights the probe pair with the highest bicor z-score among those with insignificant *A*^*MI*,*UniversalVersion*2^ z-scores (less than 1*.*9 ). Red and blue circles are selected based on all gene pairs rather than sampled ones. (B) Prediction from bicor based on Eq. 18 and observed *A*^*MI*,*UniversalVersion*2 ^are highly correlated. As in (A), one million randomly sampled gene pairs are plotted. Line y=x is added. (C) Gene expression of probe pairs highlighted by blue circles. (D) Gene expression of probe pairs highlighted by red circles.
Page two is the same analysis for ND data set using 10000 randomly selected genes rather than 3000 genes with highest variance. Click here for file

Additional file 3 **Comparison of MIC and correlation based co-expression measures.** Comparison of MIC and correlation in our empirical gene expression data sets except SAFHS. This is an extension of Figure 6. 5 best GO enrichment p-values from all modules identified using MIC and TOM are log transformed, pooled together and shown as barplots. Error bars stand for 95% confidence intervals. On top of each panel is a p-value based on multi-group comparison test. TOM outperforms MIC in all data sets except the mouse brain data. Click here for file

Additional file 4 **Compare polynomial and spline regression models to correlation or mutual information based co-expression measures in simulation.** Each point corresponds to a pair of numeric vectors *x* and *y* with length *m *= 1000. Data is simulated as in Figure 1. (A) Square root of *R*^2 ^from polynomial regression symmetrized by Eq. 5 versus absolute Pearson correlation values. The two measures are indistinguishable since the data is simulated to exhibit linear relationships. (B) *R*^2^ from polynomial regression symmetrized by Eq. 5 versus *A*^*MI*,*UniversalVersion*2^. The red line predicts *A*^*MI*,*UniversalVersion*2 ^from *R*^2^. (C-D) Same plots for spline regression models. Click here for file

Additional file 5 **Polynomial and spline regression models for estimating non-linear relationships in real data application.** In this document, we use polynomial and spline regression models to estimate non-linear relationships in real data applications. Click here for file

Additional file 6 **The relationship between module size and gene ontology enrichment p-values in 8 real data applications.** In each panel, module size (x-axis) is plotted against −log10 GO enrichment p-values (y-axis)in dots. Loess regression lines are provided to show the trend. Red and black color represent network modules constructed using TOM and *A*^*MI*,*UniversalVersion*2 ^based measures, respectively. In most data sets, the enrichment of modules defined by TOM is better than that of comparably sized modules defined by *A*^*MI*,*UniversalVersion*2^. Click here for file

Additional file 7 **Comparison of bicor, Pearson correlation and Spearman correlation based signed adjacency in 8 empirical data sets.** Each panel show the −log10 transformed 5 best gene ontology enrichment p-values of all modules identified using each type of adjacency. Error bars stand for 95% confidence intervals. On top of each panel is a p-value based on multi-group comparison test. All three types of correlation are similar in terms of GO enrichment. Click here for file
