# Supplementary material for: Comparison of co-expression measures: mutual information, correlation, and model based indices
Source: BMC Bioinformatics. 2012 Dec 9;13:328. doi: 10.1186/1471-2105-13-328 (PMC3586947; doi:10.1186/1471-2105-13-328)
Supplement: Additional file 1 — Detailed methods descriptions. In this document, we provide detail information of entropy, mutual information, likelihood ratio test statistics and p-value calculation of correlation coefficients. [file 1471-2105-13-328-S1.pdf]

Additional file 1: Methods description

Lin Song, Peter Langfelder, Steve Horvath*
* E-mail: shorvath@mednet.ucla.edu

In our article and this additional file, we use the following conventions. A random sample of m measurements of a general random variable V can be represented by a vector $v = (v_1, \ldots, v_m)$ with m components. Categorical random variables are denoted by DX, DY, DZ and numeric random variables are denoted by X, Y, Z. Correspondingly, random samples from categorical vectors are denoted by dx, dy, dz and random samples from numeric vectors are denoted by x, y, z etc.

1 Association measures between categorical variables

Consider categorical random variable DX, DY for which the frequency of the r-th and c-th level is given by $p(ldx_r), p(ldy_c)$, respectively. If dx and dy represent random samples from corresponding random variables then the approximate relationship between the observed frequencies

$$p(ldx_r, ldy_c) \approx p(ldx_r)p(ldy_c) \tag{1}$$

supports the claim that the corresponding random variables are statistically independent. A better way of saying the same thing is to use the language of hypothesis testing: if the observed frequencies $p(ldx_r, ldy_c)$ are significantly different from $p(ldx_r)p(ldy_c)$ then we reject the null hypothesis of independence between the corresponding random variables DX and DY. In this case, we say the two variables are associated with each other. To measure the difference between $p(ldx_r, ldy_c)$ and $p(ldx_r)p(ldy_c)$, one could define the sum of squared differences

$$SSq = \sum_{r=1}^{no.ldx} \sum_{c=1}^{no.ldy} (p(ldx_r, ldy_c) - p(ldx_r)p(ldy_c))^2$$

and use a permutation test to calculate a corresponding permutation p-value. But statisticians prefer a relative difference measure known as **Pearson's chi-square statistic**:

$$Pearson.chisq(dx, dy) = m \sum_r \sum_c \frac{(p(ldx_r, ldy_c) - p(ldx_r)p(ldy_c))^2}{p(ldx_r)p(ldy_c)} \tag{2}$$

The higher the value of Pearson's chi-square statistic, the stronger the statistical evidence that the **two variables are associated with each other**. To compute a p-value, one can make use of the fact that under the null hypothesis of independence between dx and dy, the chi-square statistic follows asymptotically (i.e. for large m) a chi-square distribution with $df = (no.ldx - 1) * (no.ldy - 1)$ degrees of freedom. The p-value estimation is fairly accurate if the observed cell counts satisfy $O_{rc} \geq 5$. In case of low cell counts, it is advisable to use Fisher's exact test described below.

An alternative test statistic of independence is the **likelihood ratio test (LRT)** statistic

$$LRT.statistic(dx, dy) = 2m \sum_r \sum_c p(ldx_r, ldy_c) \log \left(\frac{p(ldx_r, ldy_c)}{p(ldx_r)p(ldy_c)} \right) \tag{3}$$

where log denotes the natural logarithm (base e) and $0 * \log(any.number) = 0$ for the terms of the sum. The larger $LRT.statistic(dx, dy)$ the stronger the evidence that the corresponding random variables DX and DY are statistically dependent. Like the Pearson chi-square test statistic (Eq. 2), the LRT is an association measure that follows a chi-square distribution with $(no.ldx - 1)(no.ldy - 1)$ degrees of freedom under the null hypothesis of independence. The name "likelihood ratio test" stems from the fact that it can be derived as a likelihood ratio test of a multinomial regression model, which is a generalized linear regression model.

The p-values calculated using Pearson's chi-square statistic or the likelihood ratio test statistic are sometimes referred to as **asymptotic p-values** since they are accurate in the limit of of a large sample size m. For reasonably

large m (say observed cell counts $O_{rc} \geq 5$), the LRT and the chi-square test will lead to the same conclusions. But the approximation to the theoretical chi-square distribution for the LRT test tends to be better. For small samples **Fisher's exact test** is preferable to either Pearson's chi-square test or the likelihood ratio test. Fisher's exact test is called "exact" since the p-value can be calculated exactly, rather than relying on an approximation that becomes exact in the limit as the sample size grows to infinity, as with asymptotic statistical tests. The p-value from the test is computed as if the margins of the table are fixed which leads under a null hypothesis of independence to a hypergeometric distribution of observed cell counts O_{rc}. A drawback of Fisher's exact test is that it is computationally challenging if both $no.ldx$ and $no.ldy$ are larger than 2.

Alternatively, one can use the mutual information for contrasting the left hand side of Eq. 1 with the right hand side: the **mutual information** (MI) is defined as

$$MI(dx, dy) = \sum_{r=1}^{no.ldx} \sum_{c=1}^{no.ldy} p(ldx_r, ldy_c) \log \left(\frac{p(ldx_r, ldy_c)}{p(ldx_r)p(ldy_c)} \right) . \tag{4}$$

Here we will use a natural logarithm (base e) which implies that the MI is measured in units of "nats". It is straightforward to show that $MI(dx, dy) = 0$ if, and only if, the frequency distributions of the vectors are independent.

2 Definition of entropy among categorical variables and its relationships with mutual information

Consider the categorical random variable DX for which the frequency of the r-th level is given by $p(ldx_r)$. Then the **entropy** [?] of (the frequency distribution of) DX is defined as

$$Entropy(DX) = -\sum_{r=1}^{no.lDX} p_{DX}(ldx_r) \log(p_{DX}(ldx_r)) , \tag{5}$$

where we set $0 * \log(0) = 0$ when the probability equals 0. Consider the case when each level of DX has the same frequency (i.e. $p_{DX}(ldx_r) = 1/no.lDX$) then $Entropy(DX) = \log(no.lDX)$. One can show that this is the maximum entropy across the set of categorical vectors with $no.lDX$ distinct values. We briefly mention Shannon's defining properties of the entropy. The entropy is the only real valued function that satisfies [?]:
i) *additivity*: $Entropy(DX, DY) = Entropy(DX) + Entropy(DY)$ if DX and DY are independent,
ii) *continuity*: $Entropy(DX)$ is a continuous function of the frequency distribution $p_{DX}(ldx_r)$,
iii) *maximality with respect to constant distributions*: it takes on the maximal value when $p_{DX}(ldx_r) = 1/no.lDX$ is constant,
iv) *order-independence*: $Entropy(DX)$ is invariant with respect to the order of the frequencies $p_{DX}(ldx_r)$. The entropy is also an important concept in physics (thermodynamics) and chemistry where it is interpreted as the amount of information needed to exactly specify the state of a discrete system or the "potential for disorder" of the system. The entropy can be interpreted as a measure of uncertainty [?]: the higher the entropy, the more uncertain one is about a randomly sampled value of DX. Assume again that the vector dx (of length m) represents a random sample of DX. Then $p_{DX}(ldx_r)$ can be estimated with the observed relative frequency $p(ldx_r)$ and the $Entropy(DX)$ can be estimated with

$$Entropy(dx) = -\sum_r p(ldx_r) \log(p(ldx_r)) . \tag{6}$$

Paraphrasing [**?**, **?**], $Entropy(DX)$ quantifies the amount of surprise one should feel upon finding out the value of a single component of dx. To illustrate this, consider the extreme case where DX can take on only a single value ldx_1. In this case, $p(ldx_1) = 1$ implies that $Entropy(DX) = 0$. Clearly, one would feel zero surprise upon learning that a randomly sampled value of DX equals ldx_1. As an opposite extreme case, assume that each component of dx takes

on a distinct value, i.e. the number of levels $no.ldx = m$. In this case, $p(ldx_r) = 1/m$ and $Entropy(dx) = \log(m)$. Since all components of dx take on distinct values, one would feel maximally surprised upon learning the value of a randomly sampled component of dx.

Assume now that a second categorical variable DY is available, which takes on the values $ldy_1, \ldots, ldy_{no.lDY}$ with probabilities $p_{DY}(ldy_1)$, ..., $p(ldy_{no.lDY})$, respectively. Denote the joint probability distribution between DX and DY by $p_{DX,DY}(ldx_r, ldy_c)$. The **joint entropy** of DX and DY is defined as

$$Entropy(DX, DY) = -\sum_r \sum_c p_{DX,DY}(ldx_r, ldy_c) \log(p_{DX,DY}(ldx_r, ldy_c)).$$

Since $paste(dx, dy)$ denotes a categorical vector (of length m) with $no.ldx * no.ldy$ different levels with relative frequencies $p(ldx_r, \ldots, ldx_c)$. The joint entropy $Entropy(dx, dy)$ is defined as the entropy of the vector $paste(dx, dy)$, i.e.

$$\begin{aligned} Entropy(dx, dy) &= Entropy(paste(dx, dy)) \\ &= -\sum_{r=1}^{no.ldx} \sum_{c=1}^{no.ldy} p(ldx_r, ldy_c) \log(p(ldx_r, ldy_c)) \end{aligned} \tag{7}$$

where $p(ldx_r, ldy_c)$ is the relative frequency of observations for which dx and dy take on the values ldx_r and ldy_c, respectively. Analogously, one can define the joint entropy between three categorical vectors dx, dy, dz as follows

$$Entropy(dx, dy, dz) = Entropy(paste(dx, dy, dz)).$$

We are now ready to **express the mutual information $MI(dx, dy)$ (Eq. 4) in terms of the joint entropy between categorical vectors dx and dy**:

$$MI(dx, dy) = Entropy(dx) + Entropy(dy) - Entropy(dx, dy) \tag{8}$$

3 Empirical and Miller-Madow estimators for entropy

Consider a continuous variable X with length m. We obtain DX, the discretized version of X, by the equal-width discretization method. If the probability density p_X is known, then one can compute the true entropy $Entropy(DX)$. But in practice, p_X is unknown, and $Entropy(DX)$ must be estimated from a random sample, i.e. a discretized numeric vector $discretize(x)$. The equal-width discretization method results in a vector of relative frequencies $p = (p_1, \ldots, p_{no.bins})$ where p_r denotes the frequency of the r-th bin. Using these relative frequencies, the empirical estimator (also known as "naive", "plug-in" or "maximum likelihood" estimator of $Entropy(DX, no.bins)$ [**?**] is given by:

$$Entropy^{empirical}(DX) = -\sum_{r=1}^{no.bins} p_r \log(p_r). \tag{9}$$

The empirical estimator of the entropy tends to be smaller than the true value $Entropy(DX)$.

To counter this bias, several alternative methods have been developed. The **Miller-Madow estimator** is given by

$$Entropy^{MM}(DX) = Entropy^{empirical}(DX) + \frac{no.bins - 1}{2m}. \tag{10}$$

Note that Miller-Madow estimator does not add computational cost. Therefore, it is more favorable than the empirical estimator, and we use it as the default estimator for entropy and mutual information.

4 Estimating mutual information among (discretized) numeric variables

Many authors have proposed to use mutual information for defining association networks [?, ?, ?, ?, ?] or for clustering data [?]. While the mutual information measure is attractive for information theoretic reasons, it is challenging to estimate it in case of two continuous numeric variables X and Y. Most estimation procedures make use of the following relationship between the mutual information and the entropy $MI(X,Y) = Entropy(X) + Entropy(Y) - Entropy(X,Y)$ (Eq. 8). Thus, $MI(X,Y)$ can be defined and estimated as long as the same holds for the (joint) entropy measures. Shannon's entropy was originally defined for categorical variables but it is straightforward to define it for a continuous variable $Entropy(X) = -\int p_X(u)\log(p_X(u))du$. Although the continuous entropy shares many of the properties of the discrete entropy it has two major disadvantages. First, it may be infinitely large or negative. Second, it is in general not invariant under change of variables (e.g. monotonic transformations). Because of these reasons, researcher often prefer to define the entropy for a discretized version of the original continuous variable. Discretization turns numeric variables into categorical variables for which it is straightforward to calculate the (joint) entropies and hence the mutual information.

5 Computing p-values for correlations

In this section, we outline the approach for calculating p-value for the null hypothesis that the correlation $\rho = cor(X,Y)$ between two numeric variables X and Y is zero. The correlation coefficient between the respective random samples x and y is denoted by $r = cor(x,y)$. If either X or Y has a normal distribution, one can use a Student t-test statistic for calculating a p-value. Specifically, the Student t statistic $Z^{Student}$:

$$Z^{Student} = \sqrt{m-2}\frac{r}{\sqrt{1-r^2}}. \quad (11)$$

follows a Student T distribution with $m-2$ degrees of freedom under the null hypothesis of zero correlation. It is then trivial to calculate a p-value. It is worth repeating that the Student t-test p-value calculation only assumes that one of the variables follows a normal distribution.
