# Supplementary material for: Comparison of co-expression measures: mutual information, correlation, and model based indices
Source: BMC Bioinformatics. 2012 Dec 9;13:328. doi: 10.1186/1471-2105-13-328 (PMC3586947; doi:10.1186/1471-2105-13-328)
Supplement: Additional file 2 — Empirical analysis using large number of genes in the mouse adipose and ND data sets. Page one is an empirical analysis using all 23568 genes without restricting to 3000 genes for the mouse adipose data set. (A) Absolute value of bicor versus AMI,UniversalVersion2. One million randomly sampled gene pairs are plotted to reduce computational burden. The two measures show good monotonic relationship. The red curve predicts AMI,UniversalVersion2 from bicor. The blue circle highlights the probe pair with the highest AMI,UniversalVersion2 z-score among those with insignificant bicor z-scores (less than 1.9 ); the red circle highlights the probe pair with the highest bicor z-score among those with insignificant AMI,UniversalVersion2 z-scores (less than 1.9 ). Red and blue circles are selected based on all gene pairs rather than sampled ones. (B) Prediction from bicor based on Eq. 18 and observed AMI,UniversalVersion2 are highly correlated. As in (A), one million randomly sampled gene pairs are plotted. Line y=x is added. (C) Gene expression of probe pairs highlighted by blue circles. (D) Gene expression of probe pairs highlighted by red circles. Page two is the same analysis for ND data set using 10000 randomly selected genes rather than 3000 genes with highest variance. [file 1471-2105-13-328-S2.pdf]

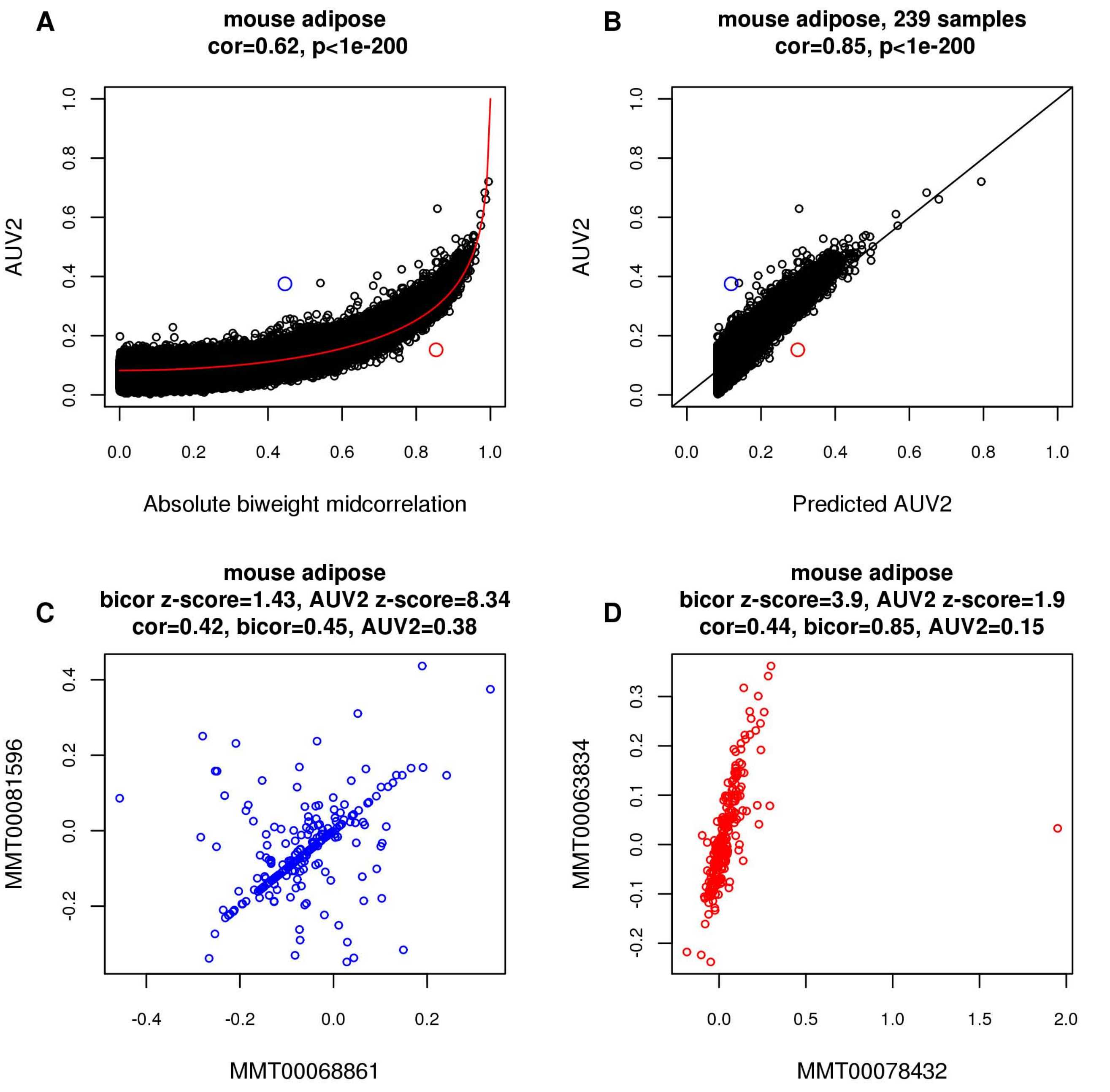

A
mouse adipose
cor=0.62, p<1e-200
AUV2
Absolute biweight midcorrelation
0.0
0.2
0.4
0.6
0.8
1.0
B
mouse adipose, 239 samples
cor=0.85, p<1e-200
AUV2
Predicted AUV2
C
mouse adipose
bicor z-score=1.43, AUV2 z-score=8.34
cor=0.42, bicor=0.45, AUV2=0.38
MMT00081596
MMT00068861
-0.4
-0.2
0.0
0.2
0.4
D
mouse adipose
bicor z-score=3.9, AUV2 z-score=1.9
cor=0.44, bicor=0.85, AUV2=0.15
MMT00063834
MMT00078432
-0.2
-0.1
0.0
0.1
0.2
0.3
0.5
1.0
1.5
2.0

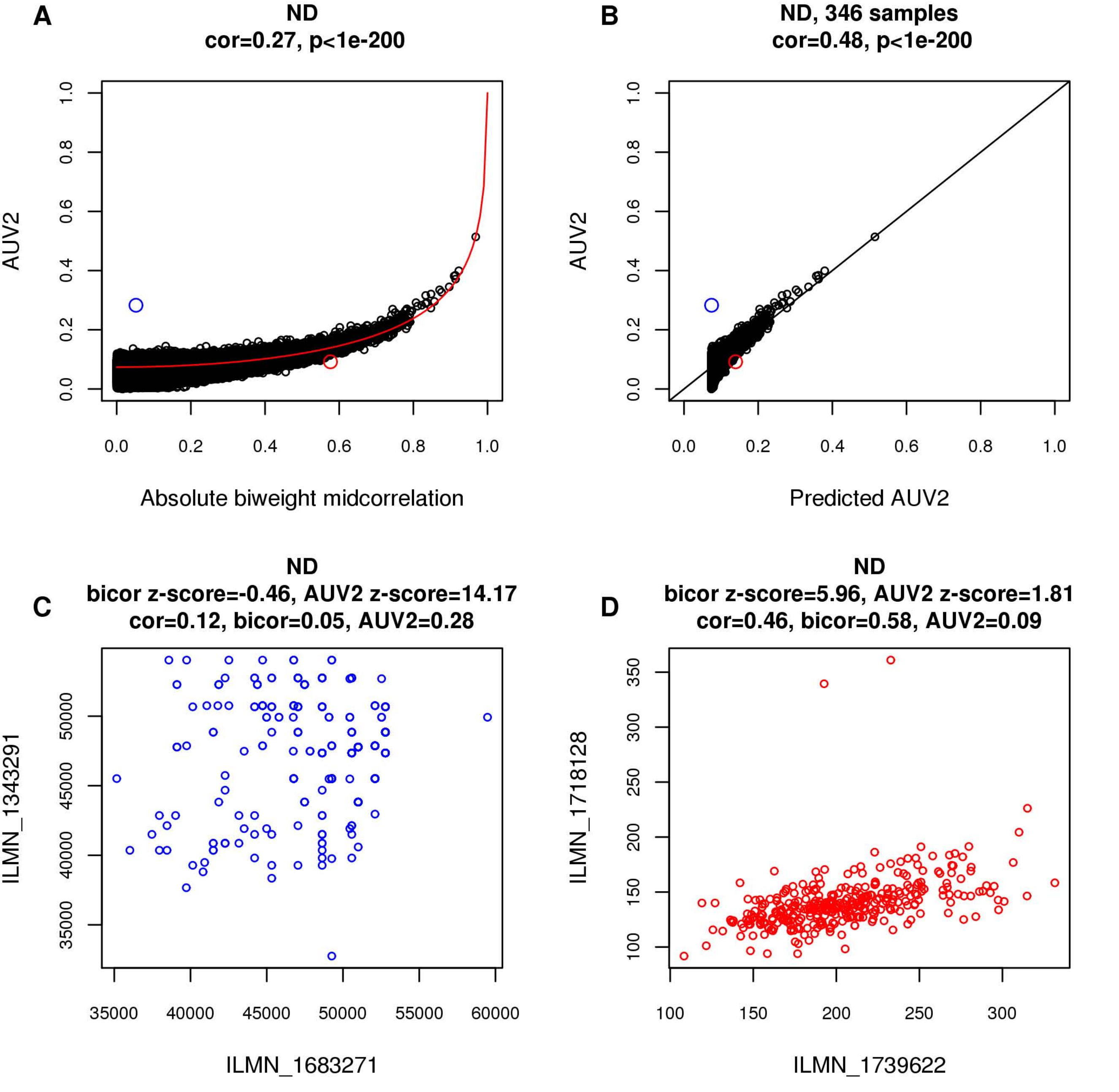
A
ND
cor=0.27, p<1e-200
AUV2
Absolute biweight midcorrelation
B
ND, 346 samples
cor=0.48, p<1e-200
AUV2
Predicted AUV2
C
ND
bicor z-score=-0.46, AUV2 z-score=14.17
cor=0.12, bicor=0.05, AUV2=0.28
ILMN_1343291
ILMN_1683271
D
ND
bicor z-score=5.96, AUV2 z-score=1.81
cor=0.46, bicor=0.58, AUV2=0.09
ILMN_1718128
ILMN_1739622
