# Supplementary material for: Comparison of co-expression measures: mutual information, correlation, and model based indices
Source: BMC Bioinformatics. 2012 Dec 9;13:328. doi: 10.1186/1471-2105-13-328 (PMC3586947; doi:10.1186/1471-2105-13-328)
Supplement: Additional file 3 — Comparison of MIC and correlation based co-expression measures. Comparison of MIC and correlation in our empirical gene expression data sets except SAFHS. This is an extension of Figure 6. 5 best GO enrichment p-values from all modules identified using MIC and TOM are log transformed, pooled together and shown as barplots. Error bars stand for 95% confidence intervals. On top of each panel is a p-value based on multi-group comparison test. TOM outperforms MIC in all data sets except the mouse brain data. [file 1471-2105-13-328-S3.pdf]

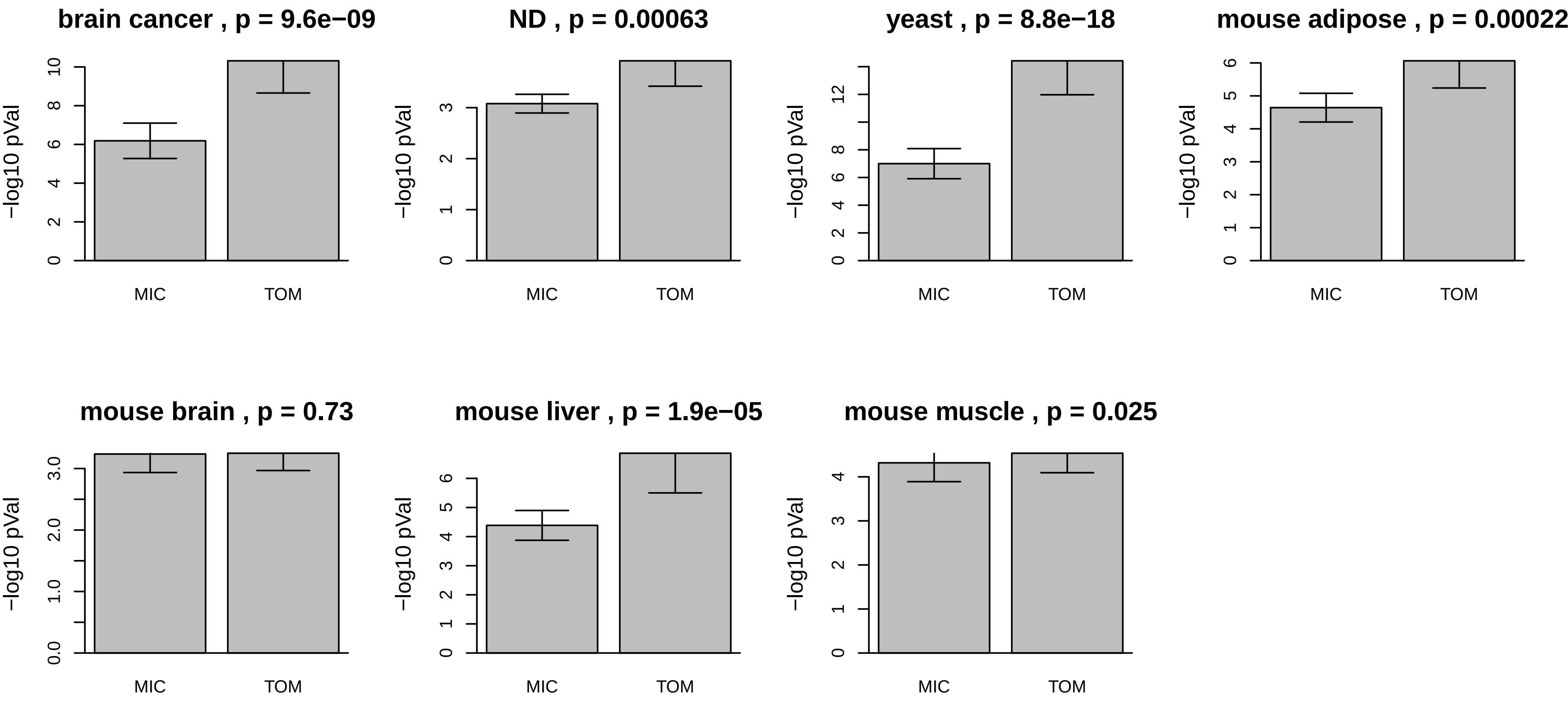
brain cancer , p = 9.6e−09
ND , p = 0.00063
yeast , p = 8.8e−18
mouse adipose , p = 0.00022
mouse brain , p = 0.73
mouse liver , p = 1.9e−05
mouse muscle , p = 0.025
−log10 pVal
MIC
TOM
