# Supplementary figures and images for: Comparison of co-expression measures: mutual information, correlation, and model based indices
Source: BMC Bioinformatics. 2012 Dec 9;13:328. doi: 10.1186/1471-2105-13-328 (PMC3586947; doi:10.1186/1471-2105-13-328)

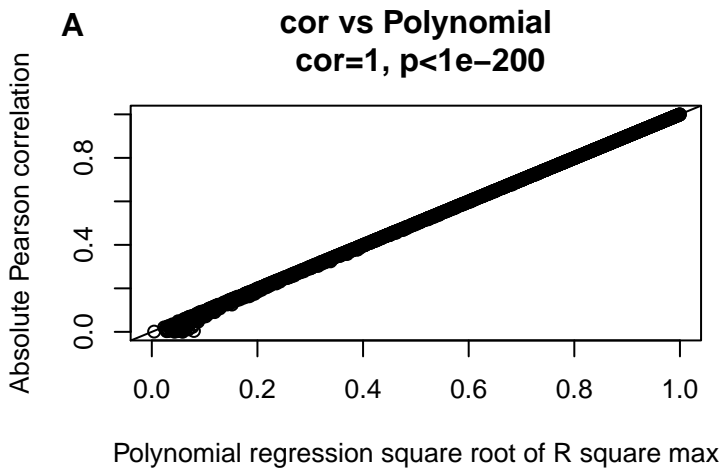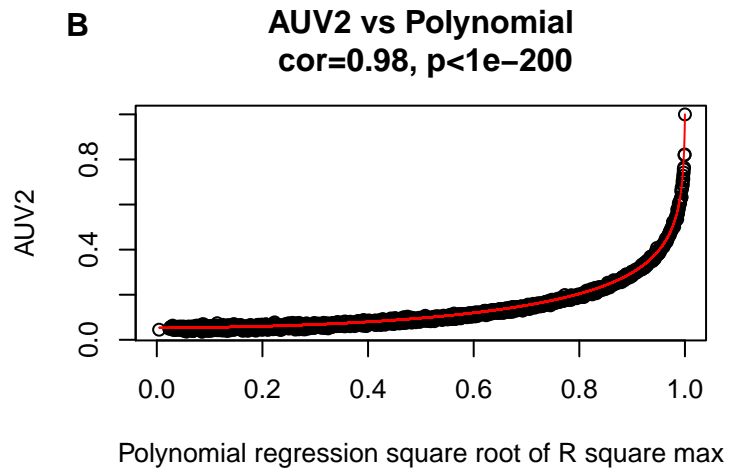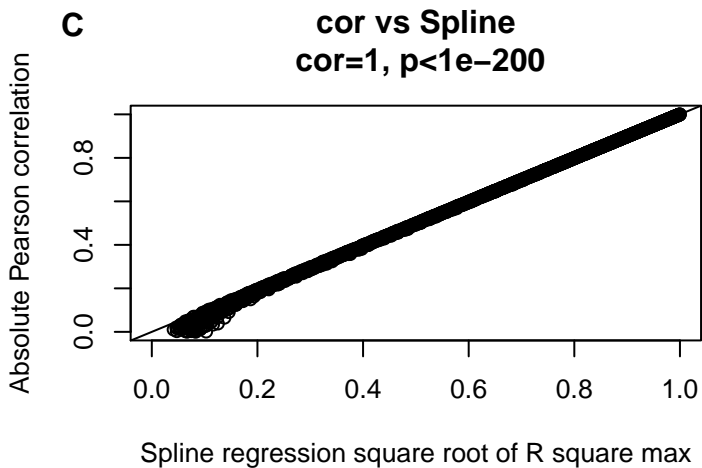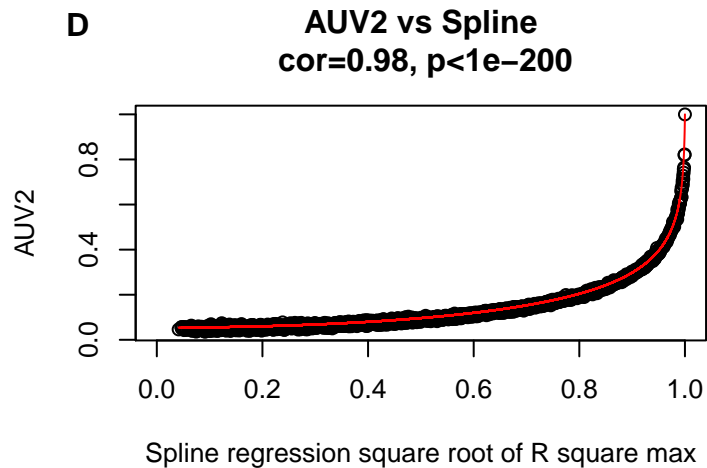

Supplement: Additional file 4 — Compare polynomial and spline regression models to correlation or mutual information based co-expression measures in simulation. Each point corresponds to a pair of numeric vectors x and y with length m = 1000. Data is simulated as in Figure 1. (A) Square root of R2 from polynomial regression symmetrized by Eq. 5 versus absolute Pearson correlation values. The two measures are indistinguishable since the data is simulated to exhibit linear relationships. (B) R2 from polynomial regression symmetrized by Eq. 5 versus AMI,UniversalVersion2. The red line predicts AMI,UniversalVersion2 from R2. (C-D) Same plots for spline regression models. [file 1471-2105-13-328-S4.pdf]

brain cancer

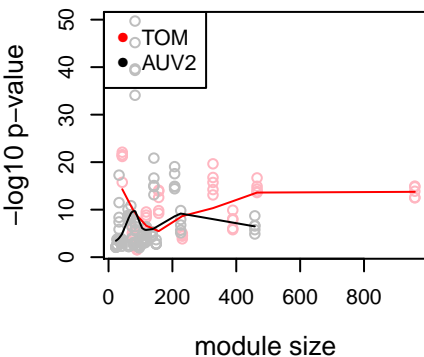

SAFHS

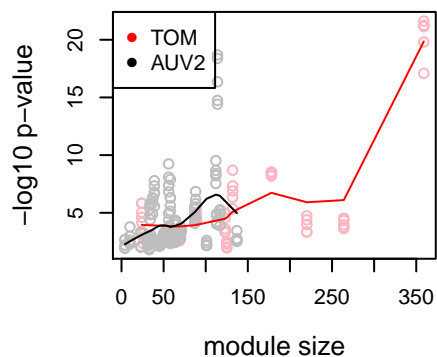

ND

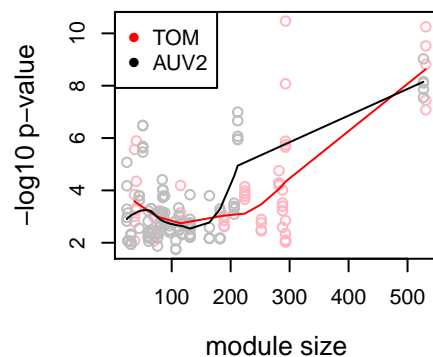

yeast

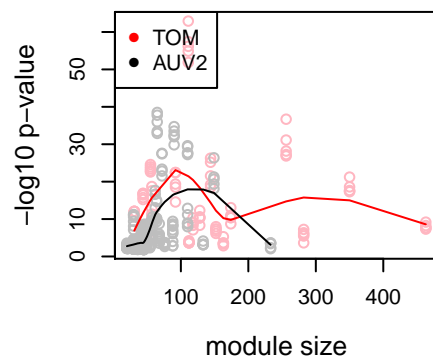

mouse adipose

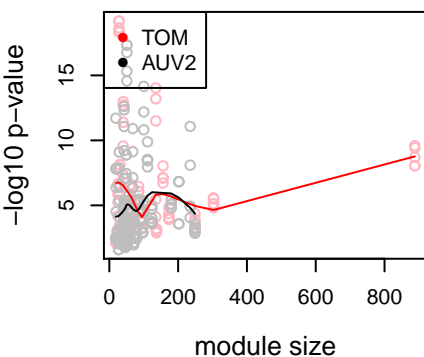

mouse brain

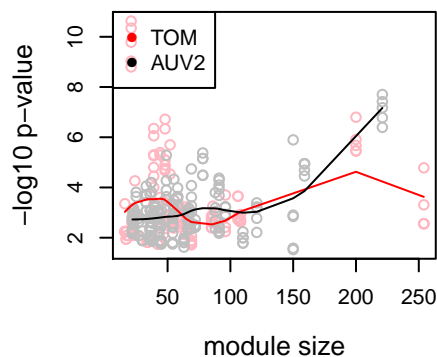

mouse liver

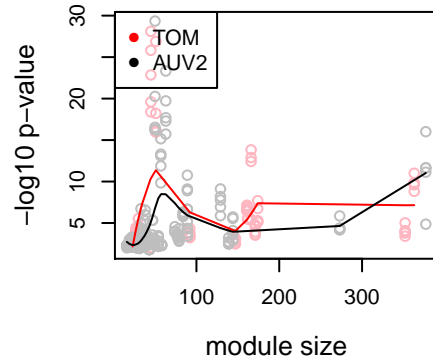

mouse muscle

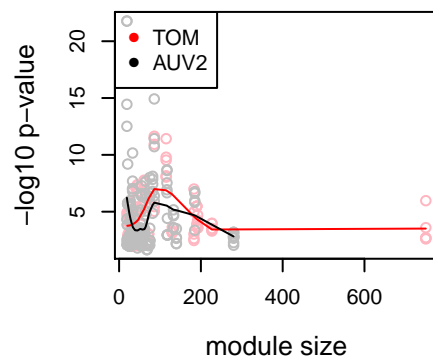

Supplement: Additional file 6 — The relationship between module size and gene ontology enrichment p-values in 8 real data applications. In each panel, module size (x-axis) is plotted against −log10 GO enrichment p-values (y-axis)in dots. Loess regression lines are provided to show the trend. Red and black color represent network modules constructed using TOM and AMI,UniversalVersion2 based measures, respectively. In most data sets, the enrichment of modules defined by TOM is better than that of comparably sized modules defined by AMI,UniversalVersion2. [file 1471-2105-13-328-S6.pdf]

**brain cancer,  $p = 0.87$**

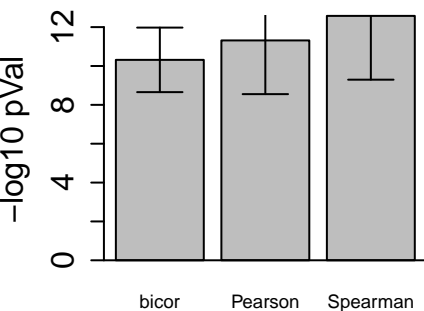

**SAFHS,  $p = 0.55$**

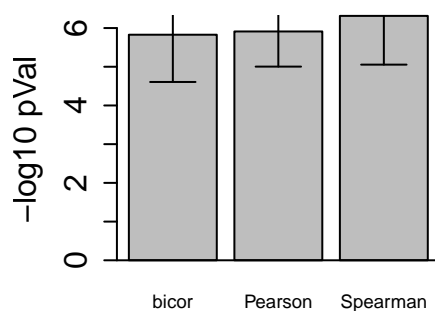

**ND,  $p = 0.49$**

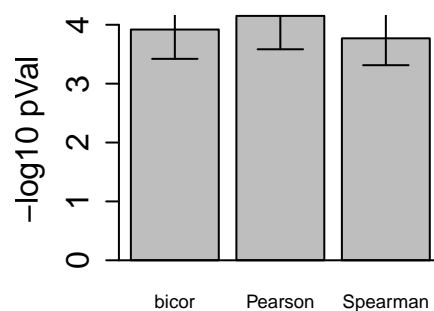

**yeast,  $p = 0.36$**

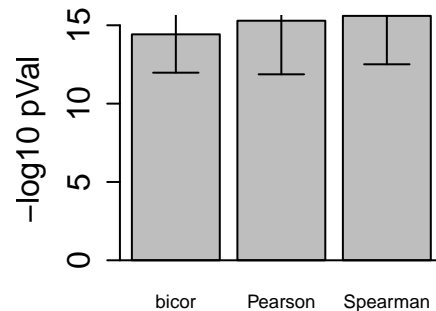

**mouse adipose,  $p = 0.36$**

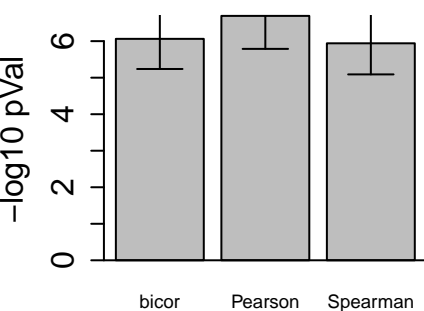

**mouse brain,  $p = 0.21$**

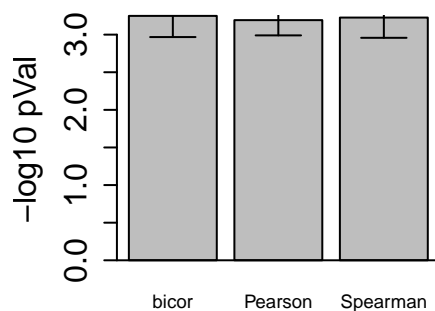

**mouse liver,  $p = 0.8$**

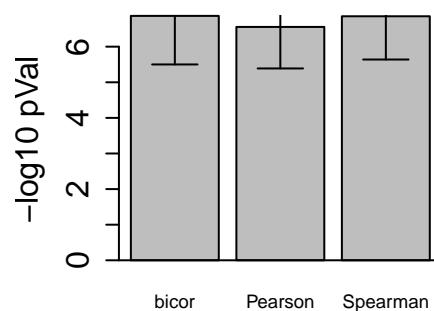

**mouse muscle,  $p = 0.96$**

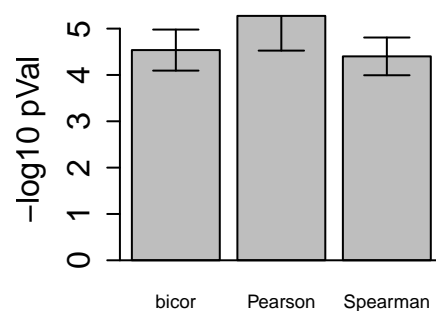

Supplement: Additional file 7 — Comparison of bicor, Pearson correlation and Spearman correlation based signed adjacency in 8 empirical data sets. Each panel show the −log10 transformed 5 best gene ontology enrichment p-values of all modules identified using each type of adjacency. Error bars stand for 95% confidence intervals. On top of each panel is a p-value based on multi-group comparison test. All three types of correlation are similar in terms of GO enrichment. [file 1471-2105-13-328-S7.pdf]
