# Supplementary material for: Comparison of co-expression measures: mutual information, correlation, and model based indices
Source: BMC Bioinformatics. 2012 Dec 9;13:328. doi: 10.1186/1471-2105-13-328 (PMC3586947; doi:10.1186/1471-2105-13-328)
Supplement: Additional file 5 — Polynomial and spline regression models for estimating non-linear relationships in real data application. In this document, we use polynomial and spline regression models to estimate non-linear relationships in real data applications. [file 1471-2105-13-328-S5.pdf]

Additional file 3: Polynomial and spline regression models for estimating non-linear relationships in real data application

Lin Song, Peter Langfelder, Steve Horvath*
* E-mail: shorvath@mednet.ucla.edu

We utilize polynomial and cubic spline regression to measure gene pairwise relationships in all 8 empirical data sets, and compare the performance with mutual information based methods.

Figure 1 compares the mutual information based adjacency $A^{MI,UniversalVersion2}$ with regression model R^2 based adjacencies for measuring gene pairwise relations in the brain cancer data set. All 6 panels show a similar pattern irrespective of regression models or R^2 symmetrization method. AUV2 and R^2 baesd adjacencies have decent positive correlations larger than that between bicor and $A^{MI,UniversalVersion2}$, indicating that both measures catch some common gene pair expression patterns that cannot be identified by corrleations. Other data sets show very similar results(Figure 2, 3, 4, 5, 6, 7, 8).

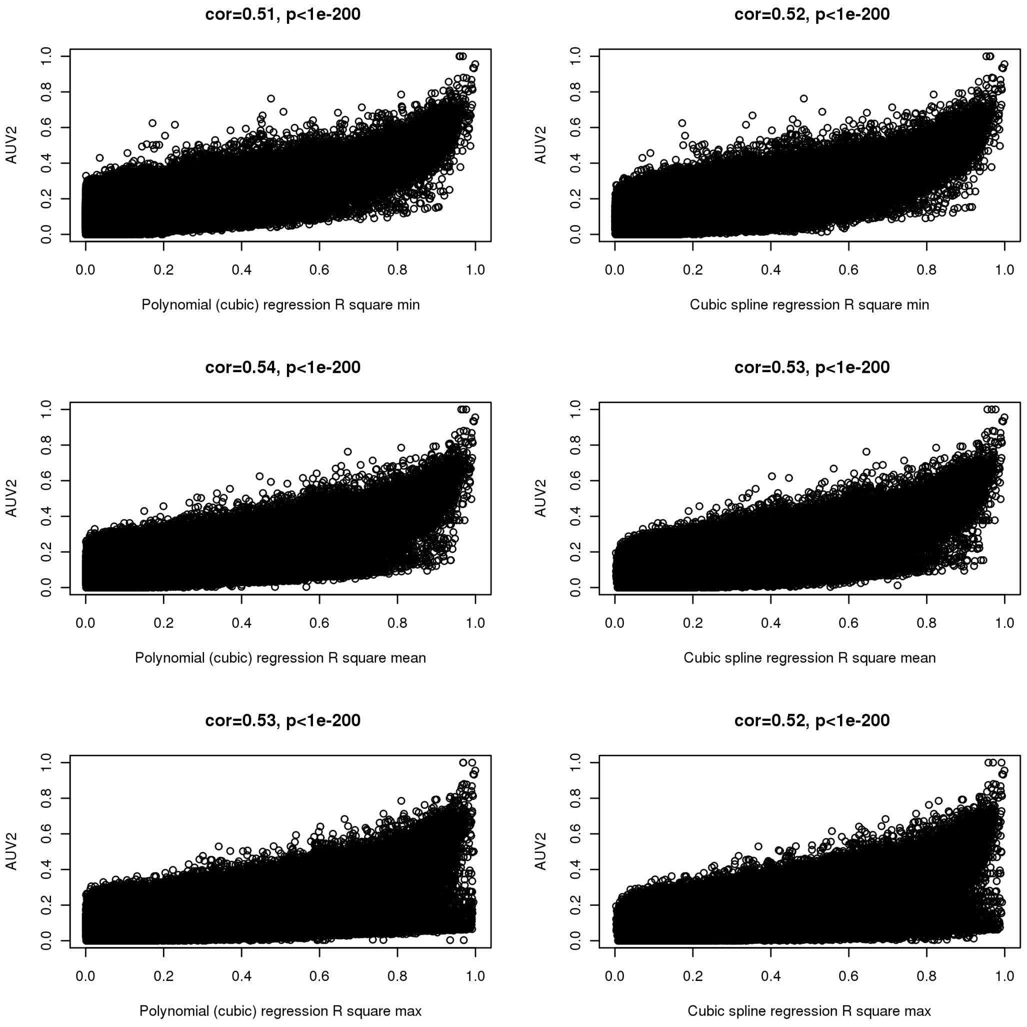

Figure 1. Brain cancer data set used to compare mutual information, polynomial and spline regression models. Coexpression of probe pairs is measured with regression models (x-axis) and mutual information $A^{MI,UniversalVersion2}$ (y-axis). The Spearman correlation and p-value of the two measures are shown on top of each panel. The left column adopts polynomial regression of degree 3 to measure probe pairwise relations on the x-axis, while the right column uses cubic spline regression. Three rows in each column represent 3 different methods to symmetrize R square matrices: taking the minimum, mean or maximum. In all 6 panels, $A^{MI,UniversalVersion2}$ and regression model R square based adjacencies are closely related, with Spearman correlation > 0.5.

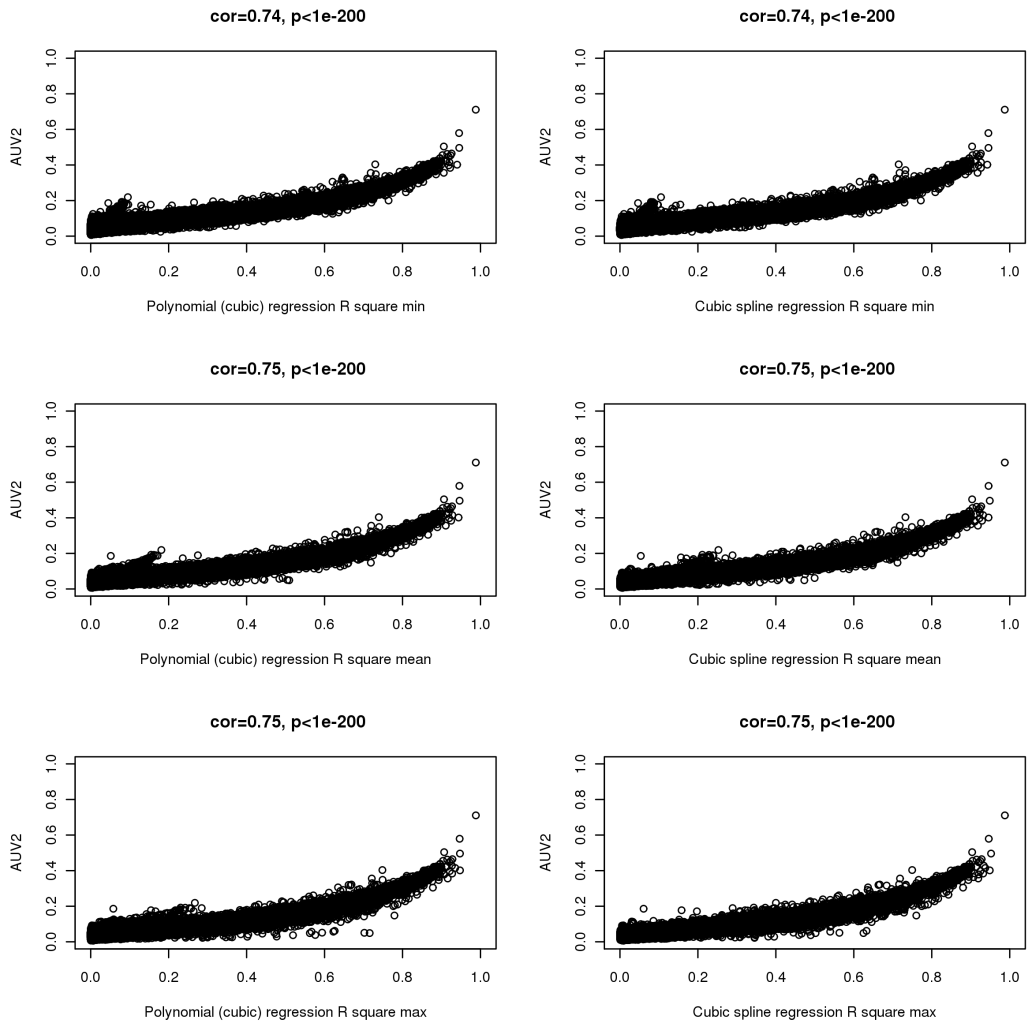

Figure 2. SAFHS data set used to compare mutual information, polynomial and spline regression models. All 6 panels exhibit clear monotonically increasing relationship between MI and regression model based measures.

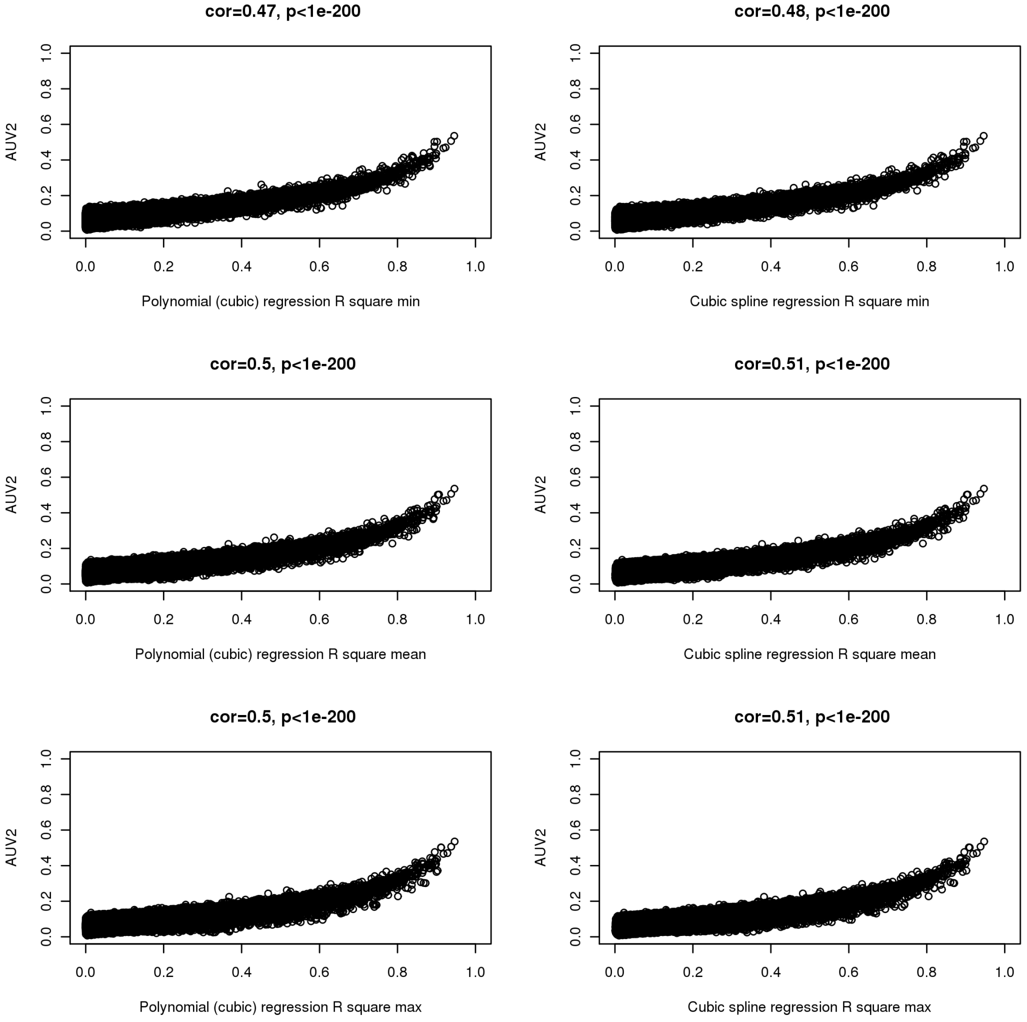

Figure 3. ND data set used to compare mutual information, polynomial and spline regression models. In all 6 panels, $A^{MI,UniversalVersion2}$ and regression model R square based adjacencies are closely related.

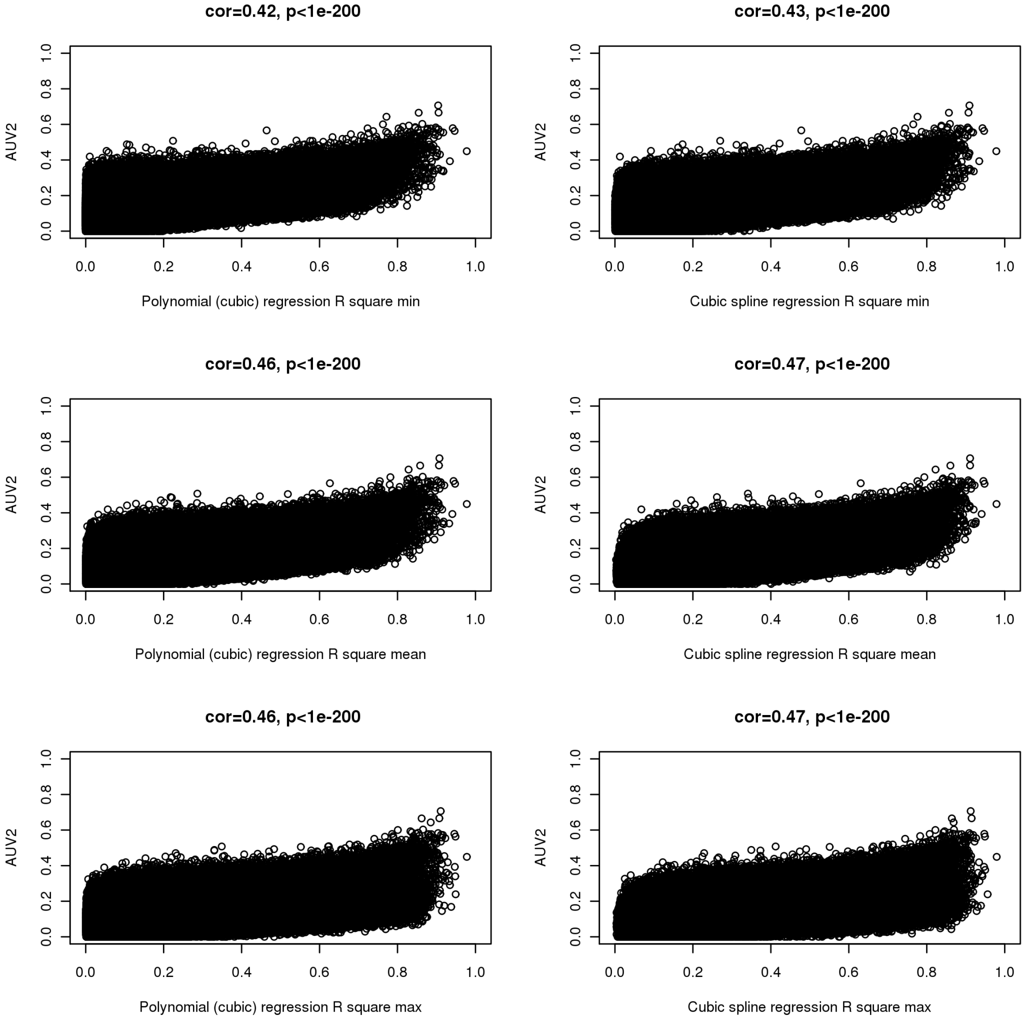

Figure 4. yeast data set used to compare mutual information, polynomial and spline regression models. In all 6 panels, $A^{MI,UniversalVersion2}$ and regression model R square based adjacencies are closely related.

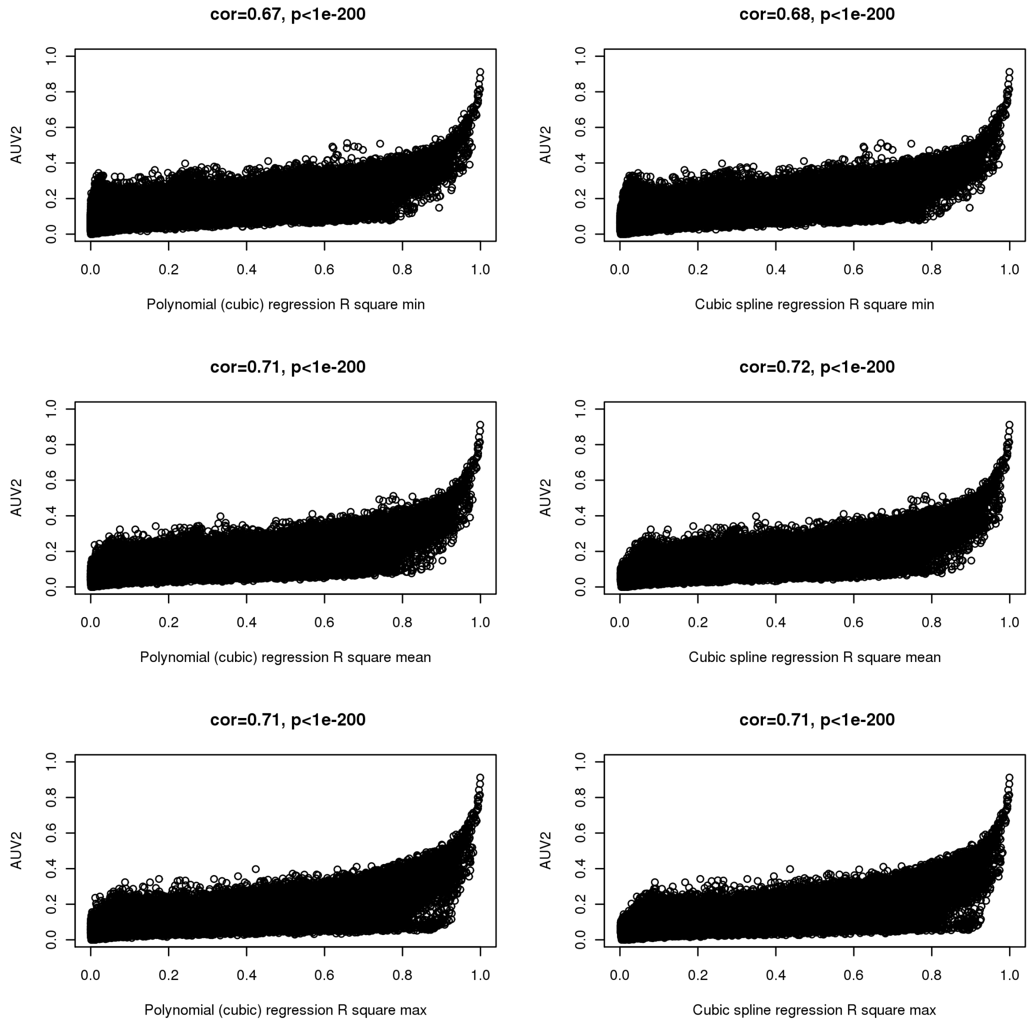

Figure 5. Mouse adipose data set used to compare mutual information, polynomial and spline regression models. In all 6 panels, $A^{MI,UniversalVersion2}$ and regression model R square based adjacencies are closely related.

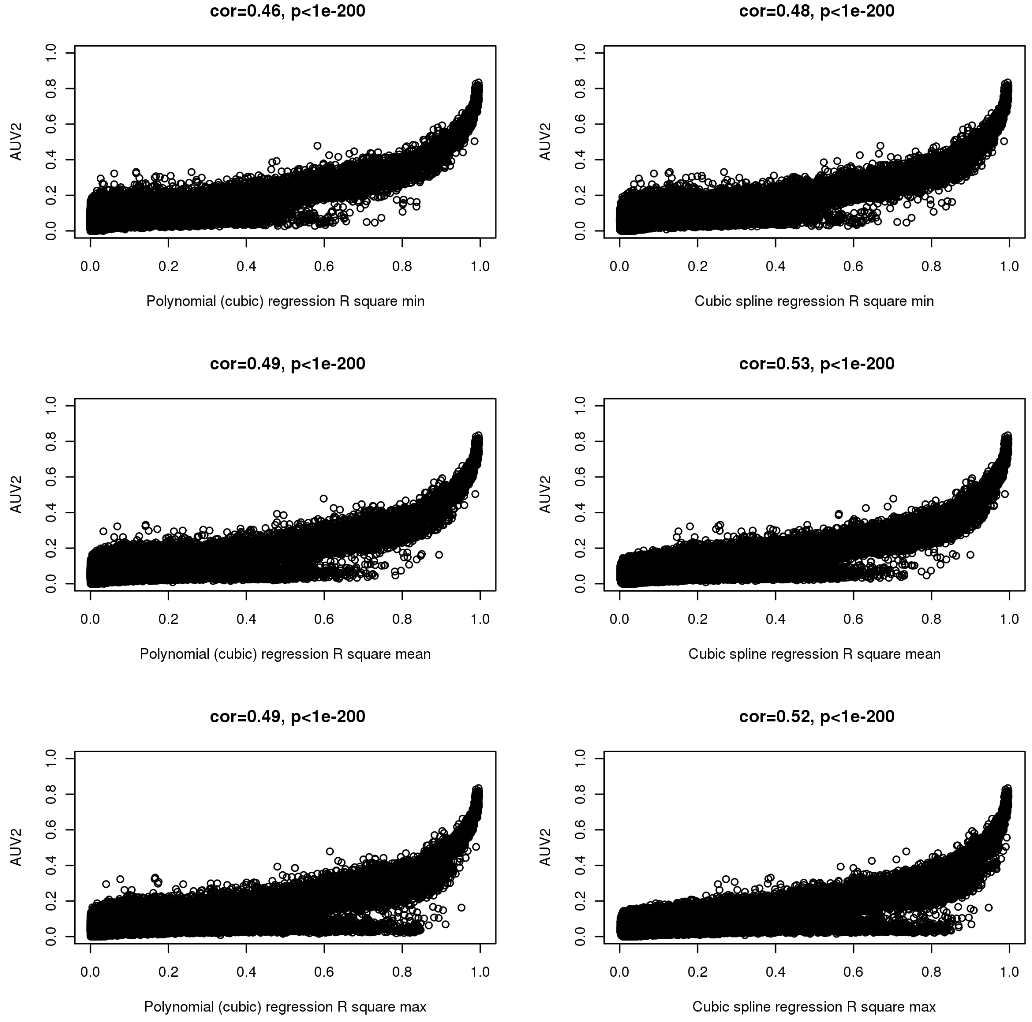

Figure 6. Mouse brain data set used to compare mutual information, polynomial and spline regression models. In all 6 panels, $A^{MI,UniversalVersion2}$ and regression model R square based adjacencies are closely related.

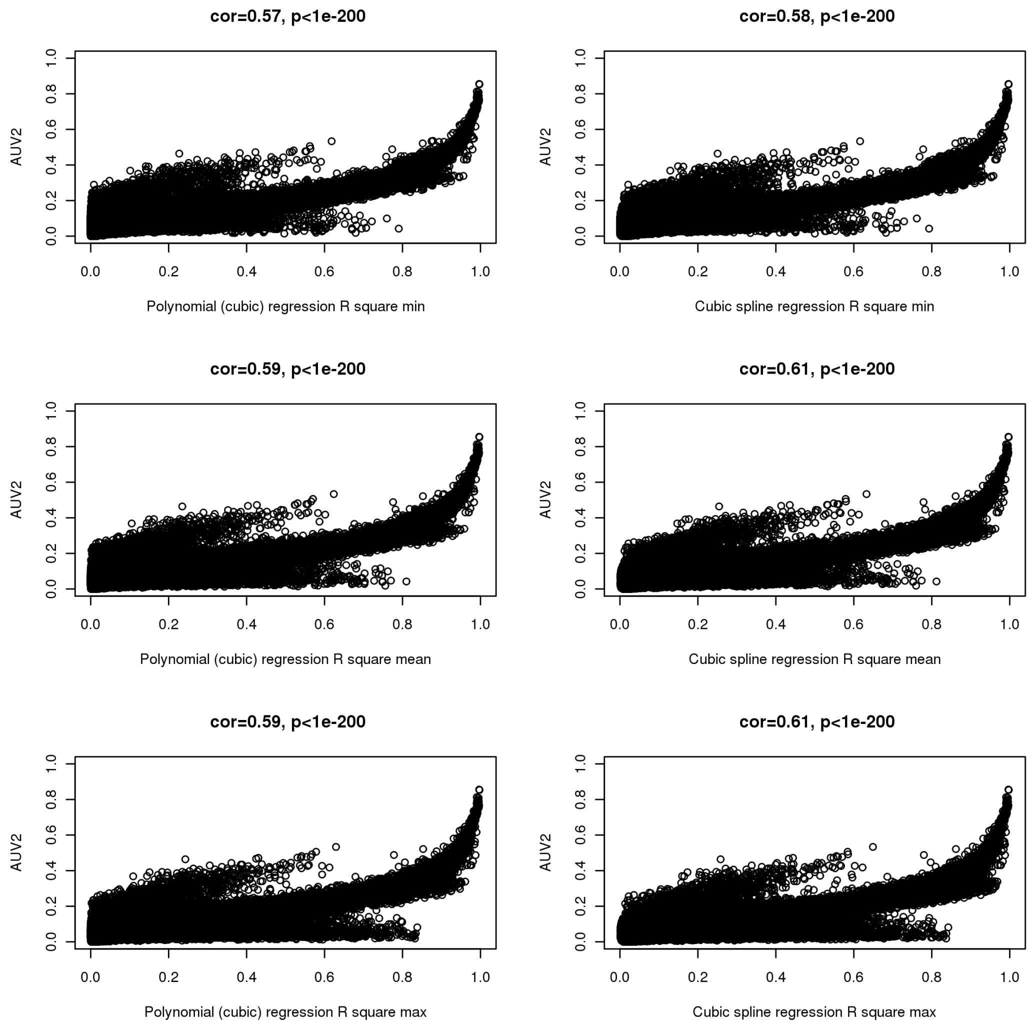

Figure 7. Mouse liver data set used to compare mutual information, polynomial and spline regression models. In all 6 panels, $A^{MI,UniversalVersion2}$ and regression model R square based adjacencies are closely related.

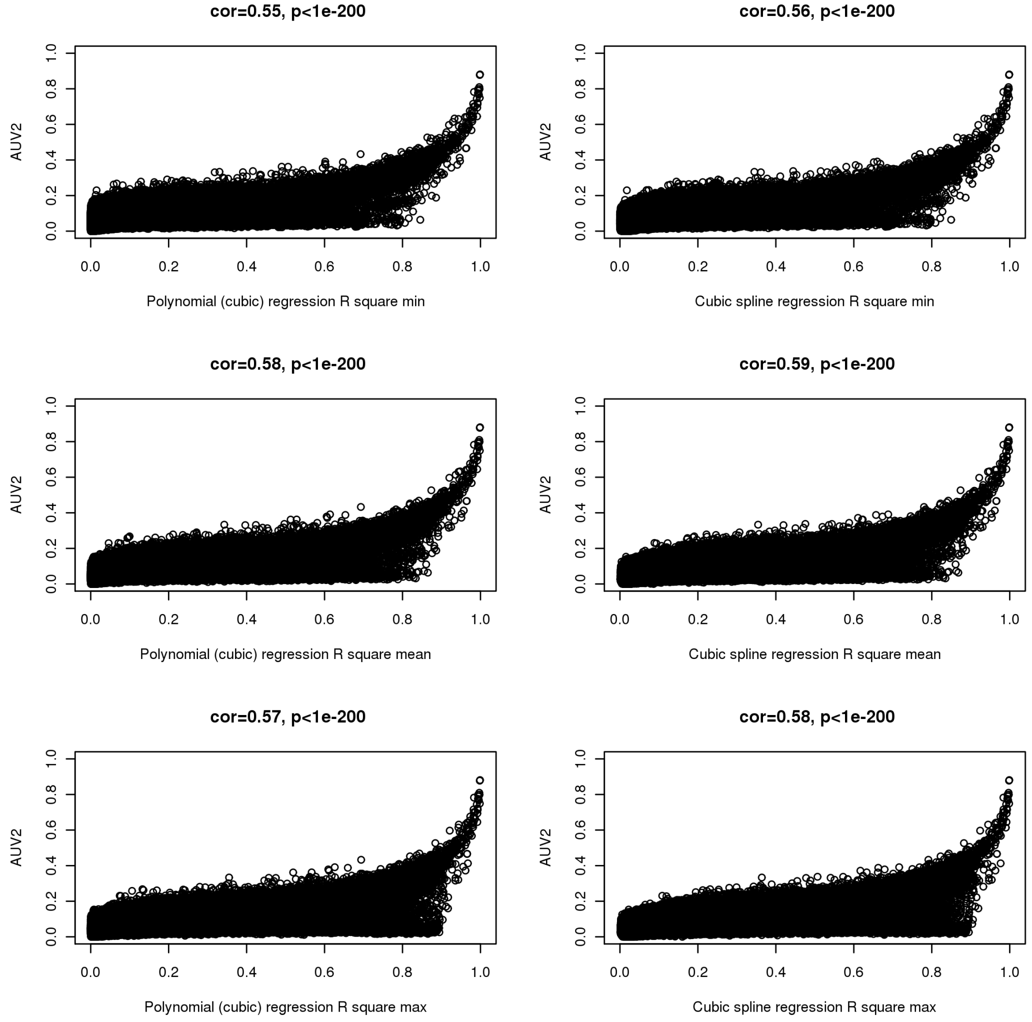

Figure 8. Mouse muscle data set used to compare mutual information, polynomial and spline regression models. In all 6 panels, $A^{MI,UniversalVersion2}$ and regression model R square based adjacencies are closely related.
